# Literature Review: Coinfection in Young Ruminant Livestock—*Cryptosporidium* spp. and Its Companions

**DOI:** 10.3390/pathogens11010103

**Published:** 2022-01-15

**Authors:** Cora Delling, Arwid Daugschies

**Affiliations:** Institute of Parasitology, Faculty of Veterinary Medicine, Leipzig University, An den Tierkliniken 35, 04103 Leipzig, Germany; daugschies@vetmed.uni-leipzig.de

**Keywords:** *Cryptosporidium parvum*, coinfections, young livestock, protozoa, zoonotic parasite, one health

## Abstract

The protozoan *Cryptosporidium parvum* is one of the major causative pathogens of diarrhoea in young ruminants; therefore, it causes economic losses and impairs animal welfare. Besides *C. parvum*, there are many other non-infectious and infectious factors, such as rotavirus, *Escherichia coli*, and *Giardia duodenalis*, which may lead to diarrhoeic disease in young livestock. Often, more than one infectious agent is detected in affected animals. Little is known about the interactions bet-ween simultaneously occurring pathogens and their potential effects on the course of disease. In this review, a brief overview about pathogens associated with diarrhoea in young ruminants is presented. Furthermore, information about coinfections involving *Cryptosporidium* is provided.

## 1. Introduction

**Figure 1:**
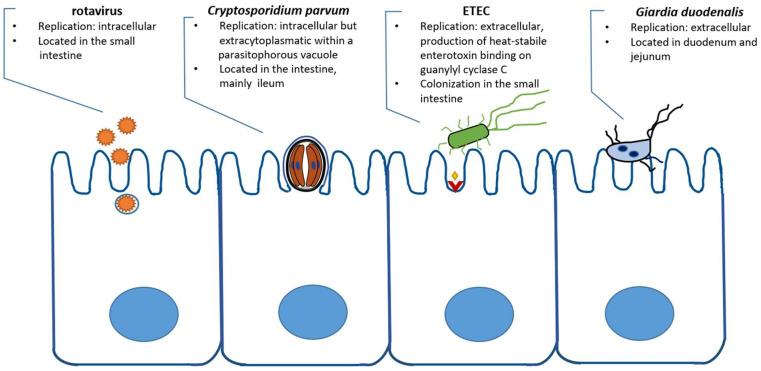
Scheme of infection localization of *C. parvum* and example pathogens in ruminants.

**Figure 2:**
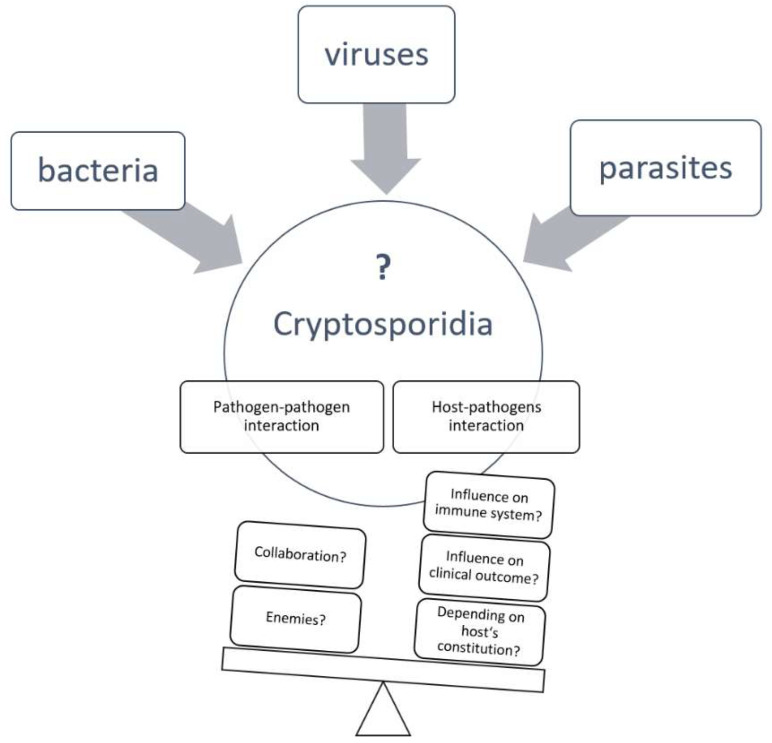
Scheme of factors that may influence a cryptosporidial infection.

In the beef and dairy industries, as well as in sheep enterprises and the goat industry, infectious diarrhoea in young ruminants is one of the biggest challenges facing economic productivity and animal welfare, leading to increased mortality rates [1,2,3]. First described in mice in 1907 by Ernest Edward Tyzzer [4], the protozoan *Cryptosporidium* is known as one of the major pathogens causing diarrhoea in young livestock, especially in calves. Formerly classified as a coccidian, this apicomplexan parasite has been transferred to the subclass, Cryptogregaria, within the Gregarinomorphea class [5,6]. After their excystation from orally uptaken oocysts, sporozoites of *C. parvum* mainly infect intestinal epithelial cells of the ileum; however, they are able to infect the gastrointestinal tract anywhere from the abomasum to the colon [2]. *Cryptosporidium* is an intracellular but extracytoplasmatic parasite (Figure 1), which reproduces asexually through two cycles of merogony and, subsequently, sexually, through gamogony. Thin-walled oocysts excyst within the host’s intestine and lead to auto-infection, whereas thick-walled oocysts are excreted to the environment and can induce infection after oral uptake [7]. Four *Cryptosporidium* spp. are regularly detected in cattle, namely *C. parvum*, *C. bovis*, *C. ryanae* and *C. andersoni*. The prevalence of these species is age-related [8,9]. *Cryptosporidium andersoni* can be detected in juvenile and adult cattle, infecting the abomasum, and it has been reported to exert an influence on milk production with no further clinical signs [10]. *Cryptosporidium bovis* and *C. ryanae* are mainly identified in post-weaned calves with no signs of clinical disease [8,9,11]. Although *C. bovis*, *C. ryanae* and *C. andersoni* have also been found in pre-weaned calves [12,13,14], *C. parvum* is mainly responsible for infection in suckling calves resulting in neonatal diarrhoeal disease [7,9,10,15]. In small ruminants, depending on the geographical region, *Cryptosporidium* species such as *C. andersoni*, *C. hominis*, *C.* *bovis* and *C. scrofarum*, as well as *C. suis*, have been described; however, *C. ubiquitum*, *C. xiaoi* and *C. parvum* are the most frequently detected species [16,17,18,19,20]. Clinical disease in lambs and goat kids due to *Cryptosporidium* infection has been mostly associated with *C. parvum* and sporadically with *C. xiaoi* and *C. ubiquitum* [18,21,22,23,24,25]. However, other non-infectious and infectious factors, including viral, bacterial and other parasitic pathogens, may contribute to *Cryptosporidium*-induced diarrhoea in young ruminants (Figure 2). Furthermore, infection with other pathogens may be modulated by active *Cryptosporidium* infection [26]. In fact, studies conducted on young, diarrhoeic livestock demonstrated that other enteropathogenic agents can be found in affected animals [27,28,29]. Although coinfections thus seem to be common in diarrhoeic calves, lambs or goat kids, information about interactions between various pathogens and the pathophysiology of coinfection is sparse [30]. This review outlines infectious pathogens that are described as agents responsible for diarrhoea in young livestock and summarizes data about their occurrence in association with *Cryptosporidium* in calves, lambs and goat kids.

## 2. *Cryptosporidium* and Viruses

### 2.1. Rotavirus

The rotavirus, belonging to the *Reoviridae* family, contains 11 segment double-stranded RNA and features a triple-layered protein capsid; it is approximately 65–75 nm in diameter [31,32,33]. In calves, this virus was first described by Mebus et al. (1969) [34]; later on, Flewett et al. (1974) suggested the name “rotavirus” [35]. Based on the genetic and antigenic characteristics of the inner capsid protein VP6, rotaviruses are classified into eight groups or species (A to H) and, in addition, new rotaviruses have recently been described in dogs, cats and bats [36,37,38]. Because of their high prevalence and pathogenicity, the most important pathogens in humans and animals of many species are the rotaviruses of group A, which are classified into G (glycoprotein) and P (protease-sensitive) types [33,38,39]. The rotavirus strains commonly detected in young diarrheic ruminants belong to group A, but in some settings, groups B and C are also frequently found and implicated in severe diarrhoea, particularly in young lambs and goats [40,41,42]. The bovine rotavirus is widespread in dairy and beef cattle throughout the world; it is described as one of the major causes of diarrhoea in calves, resulting in high morbidity and mortality as well as economic losses [29,39,43]. Prevalence rates ranging from 7% to 94% appear to depend on spatial distribution [33,39,44,45]. In lambs, the epidemiology of rotaviruses is still largely unknown; however, high morbidity (75–100%) with remarkable mortality related to neonatal diarrhoea has been described [41].

Typically, rotavirus infection takes place in calves less than 3 weeks old, but older naïve calves can also become affected in association with clinical signs [2,45,46]. After an incubation period of about 1 day, diarrhoea lasts for 1 to 2 days in an uncomplicated course of disease and much longer in case of coinfections with bacteria [2,31,46]. The diarrhoea is characterized by pasty-to-watery feces, anorexia, dehydration and prostration of the infected animal [42]. Nevertheless, shedding of rotaviruses has also been described in subclinically infected animals [28,45,47]. In recent years, many studies have been conducted to examine the prevalence of infections and coinfections with rotaviruses and *Cryptosporidium* spp. in young ruminants, especially in diarrheic calves. Both pathogens achieve high prevalence rates in calves worldwide, so it may not be surprising that these pathogens often occur as the most common coinfection [27]. Prevalence rates of coinfections have been reported in a wide range, from 2% to 85.2% [27,28,45,47,48,49,50,51]. Different prevalence rates may be explained by study design, the age of the animals and the detection method. Although no difference in the clinical manifestation between lambs infected with *Cryptosporidium* alone or together with rotavirus could be detected by Tzipori et al. (1981) [52], other authors reported the influence of coinfections on the clinical outcome. Cruvinel et al. (2020) described interactions between *Cryptosporidium* spp. and rotavirus from the 7th to 21st day of age in examined calves, leading to peaks of diarrhoea in these animals at the 15th day of life [53]. Furthermore, Göhring et al. (2014) reported that in case of concurrent infection with *Cryptosporidium* and rotavirus in calves, the fecal samples were scored more often as severe diarrhoea (62%) than the samples from singly infected calves [49]. By contrast, Lee et al. (2019) described that the fecal consistency of calves with mixed infections of rotavirus and other pathogens were very similar to those of calves with rotavirus infection only [54]. Further investigations are needed to evaluate whether *Cryptosporidium*-rotavirus coinfection may enhance the course of disease, as indicated by statistical models [51].

### 2.2. Coronavirus

Coronaviruses are large, enveloped viral particles containing a positive-sense, single-stranded RNA genome that codes for several structural proteins [55] and features a mean diameter of 100 to 120 nm with uniformly spaced, petal-shaped projections [31]. These viruses can infect a wide range of animal hosts and are divided into three antigenic groups: group 1 without hemagglutinin-esterase (HE), group 2 with HE including the bovine coronavirus and group 3 containing avian viruses [30]. The bovine coronavirus belongs to the *Coronaviridae* family within the genus *Betacoronavirus* [55] and was first described by Stair et al. (1972) and Mebus et al. (1972, 1973) in relation to neonatal calf diarrhoea [56,57,58]. Furthermore, viral infections are associated with winter dysentery in postweaned cattle and are also found in the bovine respiratory tract [59,60]. The serological prevalence is reported to be higher than 90% worldwide, suggesting that most cattle become exposed to bovine coronavirus in their lifetime [31,61]. However, the detection rate of this pathogen in diarrhoeic calves has been reported to be very low in some studies [50,62,63], while other authors have described high prevalence rates [28,59,64,65,66]. Only a small number of studies regarding the prevalence rate of bovine coronavirus in goat kids and lambs have been conducted. While the pathogen was detected rarely or not at all in young diarrheic small ruminants [67,68,69], serological prevalence has been described as ranging between 19 and 25.8% in sheep [70,71] and 41.1 and 43.1% in goats [71,72], suggesting that bovine coronavirus occurs in small ruminants with little importance in lamb and kid neonatal enteritis [69].

Bovine coronavirus can cause profuse watery diarrhoea in calves with feces containing blood and may lead to depression, reluctance to nurse, weakness and death [31,60,66,73]. Virus-associated diarrhoea occurs from one day to three or even five months of age, but mostly during the first two weeks of life [55,59,61,65]. Although the mortality due to bovine coronavirus has been described as relatively low [74,75], diarrhoea may become more severe than that caused by rotavirus, since the pathogen affects the small as well as the large intestine, with the spiral colon as the most significant zone of viral replication [59,61,76]. After an incubation period of 36–60 h, calves can develop clinical signs that usually continue for three to six days, while virus excretion may last for two or three weeks [30,31,77], and viral RNA is detectable for six weeks in the lymph nodes, ileum and colon [78]. However, bovine coronavirus also has been described regularly in asymptomatic calves [28,59]. This complicates the assessment of its role as a primary pathogen [65] and indicates that clinical manifestations are not solely dependent on the virus itself, but also on host and environmental factors such as the immunologic status of the animal, coinfections with other pathogens, and environmental temperature, since the virus is more stable at lower temperatures and reduced ultraviolet light levels [61,77].

The prevalence of coinfection with *Cryptosporidium* spp. differs between studies of diarrheic calves. In some studies, no coinfection was detected or coinfections were reported only occasionally [47,50,51,54,63], while in other studies, prevalence rates of 11.1% to 36.7% were documented [49,79]. Some authors reported an association between the presence of bovine coronavirus and diarrhoea [28,64], whereas others could not detect such a relationship [51,62]. However, Göhring et al. (2014) reported an increase in the severity of diarrhoea to 53% in the case of *Cryptosporidium*-coronavirus coinfection in calves [49]. Furthermore, Cruvinel et al. (2020) described a significant positive correlation between the occurrence of *Cryptosporidium* and coronavirus in diarrheic calves from the 6th to the 10th day of age, concluding that diarrhoea in this time frame was mainly caused by these pathogens [53].

### 2.3. Other Viruses

In recent years, a couple of new viruses were identified in cattle, and many studies were conducted to examine their influence on diarrhoea in young animals as well as their geographical distribution. Since the identification of a virus in a diarrhoeic calf is not as problematic as the understanding of its potential impact on disease, the possible role of these viruses as primary pathogens or coinfection agents is not fully understood yet [30].

The bovine astrovirus belongs to the family *Astroviridae*, which includes the two genera, *Mamastrovirus* and *Avastrovirus*. Astroviruses are small and non-enveloped, with a single-stranded, positive-sense RNA genome [80]. The bovine astrovirus was first isolated 1978, in England, from a diarrhoeic calf; it was initially considered apathogenic [81]. Later on, intestinal lesions were detected histopathologically in infected gnotobiotic calves, while these calves remained clinically normal except for the excretion of yellow and slightly soft feces [82]. In gnotobiotic lambs, astrovirus infection produced mild diarrhoea after an incubation period of 48 h [83,84]. However, until now, only a small number studies has been conducted to examine pathogenicity and distribution, as well as coinfection with astrovirus and other pathogens in ruminants in relation to diarrhoea in young animals. A study from China identified a prevalence of 46% of bovine astrovirus in diarrhoeic calves aged between 0 and 6 months; coinfection with other viruses, such as bovine coronavirus and bovine rotavirus, were present in 87.5% of cases [80]. Sharp et al. (2015) detected a high prevalence and diversity of bovine astrovirus in the feces of healthy and diarrhoeic calves (74%) in Scotland, and no association between the presence of the virus and calf diarrhoea was found [85]. Examining fecal samples from diarrhoeic calves, a study from Egypt identified the bovine astrovirus in 32% of samples, while about 37% of positive samples featured two different viruses, including bovine rotavirus and bovine norovirus [86] Due to a lack of data, it remains unclear whether the bovine astrovirus is a relevant primary pathogen, an important co-pathogen in mixed infections, or clinically irrelevant [30].

The bovine kobuvirus and the bovine enterovirus both belong to the *Picornaviridae* family, a group of non-enveloped RNA viruses that includes numerous human and animal pathogens [30]. The bovine kobuvirus (or aichivirus B) was first recognized as a cytopathic contaminant in calf sera [87] and there are several studies reporting its distribution in calves worldwide, with a prevalence ranging from 4.9 up to 77.8% [88,89,90,91,92,93]. The virus was identified in clinically healthy calves [89,90,91] as well as in diarrhoeic calves [88,93,94], but studies comparing healthy and diarrhoeic calves are limited [30]. In Brazilian diarrhoeic and non-diarrhoeic sheep, kobuvirus was also detected [89]. Although it is suggested that bovine kobuvirus can be a causative agent of diarrhoea [93], this relationship is difficult to deduce, since most studies show a lack of data concerning the incidence of other pathogens, especially bacteria and protozoans. Lee et al. (2019) detected kobuvirus in 3/164 samples from diarrhoeic calves, in which two samples also contained either *Eimeria* spp. or *Giardia* spp. [54].

The bovine enteroviruses are classified into two subgroups, E and F, and their pathogenesis and virulence in cattle are largely unknown [30]. In experimentally infected calves, clinical signs are described as varying from respiratory to enteric symptoms to reproductive disease and infertility. Nevertheless, no clinical signs were noted following acute infection, while the virus was detected in the terminal ileum, ileocecal and cecocolonic junctions, spiral colon and ileocecal lymph nodes [95]. However, some studies identified bovine enterovirus in diarrhoeic calves [94,96] and one study detected the enterovirus in feces from a diarrhoeic goat [97].

*Norovirus* and *Nebovirus* are genera of the family *Caliciviridae*; therefore, they are small, non-enveloped viruses with single-stranded, positive-polarity RNA genomes [98]. The bovine norovirus belongs to the third genogroup of noroviruses and was first described in diarrhoeic calves from England (GIII.2 Newbury-2 strain) and Germany (GIII.1 Jena strain) in 1978 and 1980, respectively [81,99,100]. Under experimental conditions, gnotobiotic calves infected with the GIII.1 Jena strain developed severe diarrhoea at 14–16 h p.i., lasting for about 3 days; severe intestinal lesions were also reported [101]. Another study demonstrated the establishment of acute intermittent, but persistent, diarrhoea accompanied by lethargy in gnotobiotic calves infected by the GIII.2 CV186-OH strain, with a lack of significant intestinal lesions [102]. High seroprevalences of bovine norovirus in cattle from Europe and the US have been reported [103,104,105]. However, the virus has been detected in diarrhoeic as well as asymptomatic cattle [106,107,108] and asymptomatic sheep [109], so its relevance in terms of diarrhoea in the field appears unclear [30]. Some studies showed prolonged virus shedding after recovery from the disease [106,107], which may explain the high prevalence rates among clinically healthy animals.

*Nebovirus* was classified recently as a new genus of *Caliciviridae* [110], after complete genome sequence analyses of two virus strains found in the 1970s in calves in the US (Bo/Nebraska/80/US) and UK (Bo/Newbury-1/76/UK) [81]. Both were identified as members of the same genus [111,112]. In gnotobiotic calves aged 17 to 60 days, anorexia, diarrhoea, and xylose malabsorption developed after infection with nebovirus [113]. Neboviruses have been reported to reach prevalence rates ranging from 3 up to 41.8% in the feces of diarrhoeic calves [114,115,116,117,118,119]. Studies including clinically healthy calves have demonstrated no virus infection or a significantly lower prevalence rate than in diarrhoeic animals [98,119], although the number of these studies is low.

Some studies demonstrated coinfections of bovine norovirus and nebovirus in calves with a prevalence ranging from 0.6 up to 10.1% [114,115,117,120]. So far, only a small number of studies have examined the presence of coinfections of bovine norovirus or nebovirus with other diarrhoea-causing agents in diarrhoeic or healthy calves. Screening fecal samples of diarrhoeic calves for bovine noroviruses and neboviruses, Karayel-Hacioglu and Alkan (2019) stated that of the infections concurrent with other pathogens, *C. parvum* spp. was the most commonly detected (46.5%) [120]. Lee et al. (2019) examined bovine norovirus in diarrhoeic calves along with 13 other causative agents of diarrhoea and detected a prevalence rate of 4.9%. The virus was found more often in calves with mixed pathogen infections than alone and was accompanied by *Escherichia coli* only or simultaneously by *E. coli*, *Eimeria* spp., *Cryptosporidium* spp. and *Giardia* spp. [54]. Fecal samples from diarrhoeic and healthy calves were examined for 11 enteric pathogens by Cho et al. (2013). The bovine norovirus and nebovirus were detected in 44.7% and 21.6% of the feces of diarrhoeic calves and in 16.3% and 1.6% of healthy calves, respectively. Bovine norovirus and nebovirus were significantly associated with diarrhoea. While nebovirus was frequently detected in feces that was also positive for bovine coronavirus, *C. parvum* or bovine torovirus, the presence of bovine norovirus was significantly correlated with the occurrence of *C. parvum* in addition to bovine rotavirus [28]. Although there was no observation of statistically synergistic interaction between pathogens regarding the severity of diarrhoea or illness in general, the authors hinted at the possibility that immunosuppressive viruses may predispose animals or humans to *C. parvum* [28].

The bovine torovirus, formerly known as Breda virus, was first detected in the USA in 1979 in diarrhoeic calves [121]. The virus is part of the genus *Torovirus* within the order *Nidovirales* [122]. Containing an elongated tubular nucleocapsid, this single-stranded RNA pleomorphic virus measures 100–140 nm × 12–40 nm in size, is kidney-shaped and features a spike-bearing envelope [121,122,123,124]. Under experimental conditions 24–72 h p.i., moderate-to-watery diarrhoea lasting three to five days is the cardinal sign of clinical infection in calves, along with a moderate increase in body temperature, depression, weakness and anorexia [124]. Diarrhoea resulting from bovine torovirus infection was also described under field conditions in calves [121,125,126,127], and in adult cattle [122,128,129]. Furthermore, the virus may also influence bovine respiratory disease [130,131]. The bovine torovirus has been found in cattle worldwide, according to epidemiological studies [125,127,132,133,134,135] and high seroprevalence was described [136,137,138,139]. Although it is also found in healthy calves, the incidence of the bovine torovirus has been found to be higher in animals with diarrhoea [64,127,133]. In one study, 56% of diarrhoeic bovine fecal samples positive for bovine torovirus were found to be subject to mixed infections with one or more enteric pathogens. This was observed more often in calves less than 6 months old than in those older than 6 months [122]. However, virus shedding was not consistently associated with any other agent observed in that study. Another study detected the bovine torovirus in 5.2% of examined fecal samples from diarrhoeic and nondiarrhoeic calves; most of the virus-positive samples contained one or more additional pathogens, including bovine coronavirus, *E. coli*, *Giardia* spp., cryptosporidia, *Eimeria zuernii/bovis*, *Klebsiella* spp., rotavirus and *Clostridium perfringens* were detected [64]. In a study of diarrhoeic Korean native calves, torovirus was detected in 6.7% of fecal samples and was mostly found in calves also infected with other pathogens, such as *Cryptosporidium* spp. or *E. coli* [54]. Nogueira et al. (2013) found bovine torovirus in 6.25% of diarrhoeic fecal samples. One sample contained bovine torovirus alone; the others were also positive for *Cryptosporidium* spp. or bovine coronavirus. These findings led to the suggestion that this virus is potentially a primary enteric pathogen in cattle and may also play a synergistic role in mixed infections [129].

## 3. *Cryptosporidium* and Bacterial Infection

### 3.1. Escherichia coli

First isolated in 1885 from a child [140], *E. coli* is known to commonly inhabit the gastrointestinal tract of humans and animals, with some strains being harmless commensals and others acting as major pathogens [141]. Belonging to the family Enterobacteriaceae, the gram-negative, flagellated and facultative anaerobic bacterium is classified into six diarrhoeagenic pathotypes, as follows: enterotoxigenic *E. coli* (ETEC), enteropathogenic *E. coli* (EPEC), Shiga toxin-producing *E. coli* STEC (i.e., enterohaemorrhagic *E. coli* [EHEC]), Shigella/enteroinvasive *E. coli* (EIEC) enteroaggregative *E. coli* (EAEC) and diffusely adherent *E. coli* (DAEC) [142,143,144]. *E. coli* pathotypes are characterized by O (lipopolysaccharide, LPS) and H (flagellar) antigens defining serogroups or serotypes [143]. STEC is determined by the presence of Shiga toxin 1 or 2 gen, while EHEC is a subset of STEC [144]. EPEC and EHEC are both able to induce characteristic intestinal histopathology, known as attaching and effacing lesions [143]. Attaching and effacing *E. coli* (AEEC) strains have been reported in diarrhoeic calves, lambs and goats [145,146,147,148,149,150,151], as well as in healthy animals [152,153]. Although the prevalence of STEC, EPEC and EHEC in diarrhoeic and healthy calves has been found to be high, no association with diarrhoea has been described [154]. It has been suggested that calves and small ruminants may play an important role as zoonotic reservoirs for these human pathogenic *E. coli* [150,154,155]. La Ragione et al. (2006) demonstrated that experimentally infected lambs shed higher amounts of EHEC after pre-inoculation with *C. parvum*, suggesting that a better understanding of their relationship would lead to more effective intervention strategies in the field [156]. While EAEC, DAEC and EIEC are less frequently reported in cattle [154], another pathotype, necrotoxic *E. coli* (NTEC), which produces either cytotoxic necrotizing factor 1 (CNF1) or CNF2, has been associated with disease in animals and humans [143]. NTEC has been isolated from diarrhoeic [157,158] and healthy cattle [153,159,160,161]. Thus, its major role as an enteropathogen in young ruminants is questionable. Nevertheless, NTEC may be an opportunistic pathogen waiting for suitable circumstances [158].

ETEC is able to produce either heat-labile (LT) or heat-stable (ST) enterotoxin and possesses several colonization factors for adherence to the intestinal epithelium [144]. F4, F5, F6, F17 and F41 are fimbrial adhesins responsible for adherence, while F5 (K99) and F41 have been reported to be highly related to the presence of diarrhoea in calves [154]. In beef and dairy calves, ETEC has been identified as the major cause of neonatal diarrhoea during their first 4 days of life, whereas diarrhoea is rare in older calves and adult animals [2]. Nevertheless, in some studies that examined the concurrent occurrence of several pathogens in young diarrhoeic ruminants, only small numbers of *E. coli* (K99) in terms of single or coinfection with *C. parvum* were detected [28,49,50,62,162,163]. An explanation for this could be the short period of time in which *E. coli* is shed or the use of vaccination, resulting in a low prevalence, although its incidence might be relatively high [62]. However, other authors discovered a higher prevalence of ETEC and coinfection with *Cryptosporidium* spp. ranging from 12% to 27.8% [45,54,79,164]. Tzipori et al. (1981) examined the coinfection of *C. parvum* and ETEC in experimentally infected lambs. Although ETEC was found in the feces of these animals, no mucosal colonization by the organisms was detected in any part of the intestine and bacterial infection did not aggravate the clinical response to *Cryptosporidium* [52]. Since experimental studies considering coinfection with *E. coli* and *Cryptosporidium* related to the clinical outcome of the disease are rare, the relevance of *E. coli* during the exposure of calves to cryptosporidia is difficult to assess. Nevertheless, the synergistic effects of coinfection were shown for concurrent infection with *E. coli* and rotavirus in experimentally infected calves [165,166,167,168,169]; this may also apply to *E. coli* and particularly ETEC in mixed infection with cryptosporidiosis under certain conditions. However, sufficient data to prove this assumption are lacking.

### 3.2. Clostridium

Clostridia are anaerobic, gram-positive, spore-forming, rod-shaped bacteria [170]. This ubiquitous and soil-borne bacterium is part of the gastrointestinal microbiota of healthy and diseased animals and humans [170,171]. Nevertheless, members of this genus are widely recognized as enteric pathogens in humans and animals [172]. In ruminant livestock gastrointestinal diseases caused by clostridia are common and have been described as hemorrhagic enterocolitis, enterotoxemia, pulpy kidney disease, overeating disease, braxy (bradsot), struck, lamb dysentery, enterotoxemic jaundice, yellow lamb disease, clostridial abomasitis and clostridial enteritis [173]. Except for braxy (caused by *Clostridium septicum*), all of these disease conditions are caused by subtypes of *Clostridium perfringens* [173], which was first isolated by William H. Welch from the autopsy of a 38 year-old man in 1891 and originally named *Bacillus aerogenes* [170,174]. Five defined types, or genotypes, of *C. perfringens* exist: A, B, C, D and E. They are identified by their production of lethal toxins, namely *C. perfringens* alpha, *C. perfringens* beta, epsilon and *C. perfringens* iota [173]. Recently, the addition of two new toxinotypes, namely F and G, has been suggested, based on the ability to produce *C. perfringens* enterotoxin, or NetB toxin [175]. Most common in warm-blooded animals are the strains of type A, which are able to cause wound contamination, anaerobic cellulitis, gas gangrene and enteric diseases [172]. In calves, lambs and kids, enteric disease caused by the type A strain can manifest in the form of enterotoxemia, necrotizing enterocolitis, haemorrhagic enteritis, abomasal necrosis and sudden death [176,177,178,179,180,181]. In neonatal ruminants, *C. perfringens* type B causes acute hemorrhagic enterocolitis, generally known as lamb dysentery in lambs; it is restricted to lambs less than 21 days of age, or calves younger than 10 days, respectively [173]. While the primary sign is death in peracute cases, acute cases are characterized by reduced feed uptake and severe abdominal pain accompanied by bloody diarrhoea, coma and death less than 24 h after onset [172,182,183]. The clinical symptoms of infection with *C. perfringens* type C are similar to those of type B strains, occurring in lambs and calves and, rarely, in goats [173,182,184,185,186]. Both types are able to express beta toxin, which is susceptible to proteolytic destruction by trypsin; therefore, neonatal ruminants are generally at higher risk of disease [172,173,185]. While *C. perfringens* type D infection causes enterotoxemia in small ruminants of all ages, clinical enteritis is rare in calves and lambs, but consistently found in goats [173,187,188]. The occurrence of *C. perfringens* type E is rarely described in neonatal calves, but the infection seems not to be uncommon and is able to cause hemorrhagic enteritis and sudden death [185,189,190]. Infection has also been described in kids with severe diarrhoea and sudden death [191].

Whether *C. difficile* is also a pathogen associated with diarrhoea in calves, or whether calves rather act as reservoirs for human disease, is not completely understood yet, since this agent has been detected in diarrhoeic as well as in healthy calves [192,193,194,195,196]. However, although clostridia are known to be causative agents of diarrhoeic disease in young ruminants, information about their influence on coinfections is limited. Only a small number studies that examine the occurrence of *Clostridium* spp. or its toxins in combination with other viral or parasitic pathogens have been conducted. Singh et al. (2018) investigated clostridia infection mixed with EPEC, bovine rotavirus and bovine coronavirus in diarrhoeic kids and reported prevalences of 11.8%, 3.8% and 2.1%. The authors detected an incidence of 75% type A and 25% type D in *C. perfringens*-positive samples [188]. While examining a broad range of pathogens in samples from diarrhoeic and asymptomatic calves, Cho et al. (2013) did not find a *Clostridium perfringens* toxin β gene, indicating that neither *C. perfringens* type B nor type C was present [28]. By contrast, two other studies demonstrated a high prevalence of *C. perfringens* and high numbers of coinfection with *Cryptosporidium* spp. in calves [62,164]. Unfortunately, no further investigations regarding the types of *C. perfringens* or the presence of toxins were undertaken, limiting conclusions as to the respective participation in the development and course of diarrhoea in these studies. Lee et al. (2019) detected *C. difficile* in 9.8% of diarrhoeic singly or coinfected calves by confirming the presence of the *tcdB* toxin gene, concluding that clostridia was one of the main causative agents [54].

### 3.3. Salmonella

The gram-negative and facultative anaerobe bacterium *Salmonella* was first isolated from the intestines of pigs by Theobald Smith in 1855. It is classified into two species, *Salmonella enterica* and *Salmonella bongori*, based on differences in their 16S rRNA sequence analysis [197]. *Salmonella enterica* can be further divided into six subtypes. *S. enterica* subspecies *enterica* is the most relevant in dairy cattle [198], as well as in other mammals. Around 99% of *Salmonella* infections in humans and warm-blooded animals are caused by this subtype [197]. Furthermore, more than 2500 serovars based on somatic (O), flagellar (H) and capsular (Vi) antigens have been identified; however, only a small number are of clinical relevance in cattle [198]. In calves, mostly subgroups of *S. enterica* serovars such as *S.* Typhimurium, *S.* Newport and *S.* Dublin (a specific bovine-adapted serotype) are important causes of diarrhoea [199,200]. The diarrhoea can appear watery-to mucoid-with fibrin and blood and is often accompanied by elevated temperature and refusal to eat or drink [201,202,203]. Infected calves are often septicemic, in addition to suffering from enteric disease, resulting in severe clinical signs [199]. A wide range of *S. enterica* serovars is able to infect sheep [204,205]. Among others, *S.* Typhimurium and *S.* Dublin have been implicated as etiologic agents of diarrhoea in lambs [206]. While the host-specific *S.* Abortusovis leads to abortion and mortality in lambs, *S. enterica* ssp. *diarizonae* is also considered to be host-specific and is able to produce intestinal and extra-intestinal infections, mostly without clinical disease [204,205,207,208]. Field studies reported infection with *S.* Typhimurium, *S.* Chester, *S.* Saintpaul, *S.* Adelaide, *S.* Muenchen and *S.* Singapore in goats [209,210,211]. Experimental infection with *S.* Typhimurium in goats resulted in mild-to-severe clinical symptoms, including diarrhoea, anaemia, pyrexia, progressive weakness and loss of body weight [212,213].

Coinfection with *Salmonella* spp. and cryptosporidia or rotavirus has been reported in calves; however, the prevalence of salmonellae was low, ranging from 0.5% to 1.9% [45,48,51,54,214]. By contrast, Cho et al. (2013) described a prevalence of 9.0% of *Salmonella* spp. in diarrhoeic calves, commonly detected in fecal samples that were also positive for rotavirus [28]. In another study, it was reported that 40% of *C. parvum*-positive fecal samples were also positive for *Salmonella* spp. [164]. Muñoz et al. (1996) examined fecal samples from diarrhoeic lambs and goat kids. *Salmonella enterica* ssp*. arizonae* was isolated from only one goat kid that was also infected with cryptosporidia [68]. In 3 of 21 examined diarrhoeic calves, *S.* Typhimurium was diagnosed together with either coronavirus or cryptosporidia and adherent bacteria or *E. coli* in fecal or intestinal tissue samples. In two of three cases, *S.* Typhimurium was assumed to be the principal cause of diarrhoea [215]. Examining the efficacy of halofuginon for the prevention of natural cryptosporidiosis in calves coinfected with rotavirus and *S.* Typhimurium, Almawly et al. (2013) concluded that the use of this drug did not improve the clinical outcome and anti-*Cryptosporidium* activity was not fully preserved in the presence of coinfection. The authors hint at the possibility that there could be a dilution or a reduced transit time of halofuginon because of the presence of the other pathogens and their impact on increased fluid content and intestinal motility [216].

## 4. *Cryptosporidium* and Other Parasites

### 4.1. Protozoa

#### 4.1.1. Giardia

*Giardia duodenalis*, a protozoan flagellate parasite, features a direct life cycle and its cysts, which are excreted in the host’s feces, are immediately infectious [217]. This parasite features a global distribution and a broad range of potential mammalian hosts. Because of the varying clinical outcomes of *G. duodenalis* infection in several studies, the pathogenicity of *Giardia* has been debated for quite some time. It has been suggested that the clinical outcomes of giardiasis are related to strain variability, host nutritional status and mucosal immune response, as well as the composition of microbiota, immune modulation by *Giardia* and the presence of coinfecting enteropathogens [218]. *Giardia* is highly prevalent in livestock worldwide, with an individual prevalence ranging from 9 to 73% and a farm prevalence varying between 45 to 100% in cattle [219]. The parasite is able to cause diarrhoea, reduced weight gain and the impairment of feed efficiency in calves, goat kids and lambs and can also lead to reduced lamb carcass quality and livestock productivity [219,220,221,222,223]. Within the *G. duodenalis* complex, eight groups of genetically related strains (assemblages A to H) are strongly supported, of which two (A and B) are zoonotic [224]. In addition to both human relevant assemblages A and B, assemblage E (hoofed livestock assemblage) has also been described in ruminant livestock [219,225,226,227].

In recent years, many studies have been conducted to evaluate the epidemic situation of *G. duodenalis* in co-existence with *Cryptosporidium* spp. in ruminant livestock all over the world, demonstrating that the prevalence of both parasites varies between regions and animal species [11,17,225,226,228,229,230]. Although several studies indicate that *Giardia* mainly occurs in younger ruminants and can be detected as early as in the first week of life [228,231,232,233], there are also reports about giardiasis in adult animals that are mostly considered to be asymptomatic [234,235]. The peak of *Giardia* prevalence and/or cyst excretion differs between the studies conducted in calves, ranging between an age of 12 weeks to 20 weeks [236,237], 4 to 12 weeks [50,229,238,239], or even in animals at 2 weeks of age [240]. Although several studies on cattle, sheep and goats demonstrated the co-existence of *Giardia* and *Cryptosporidium* in farms or herds, many of these studies did not detect coinfection in animals [17,225,226,235,241]. Nevertheless, several authors reported coinfection in ruminant livestock [216,228,230,239,240,242,243,244,245,246], even though coinfection was not as frequent as *Giardia* mono-infection, a fact leading to the suggestion that coinfection with *Cryptosporidium* and *Giardia* in cattle is uncommon [232]. In cattle, *Cryptosporidium* causes disease in calves younger than 1 month [220]. Different age preferences of *Giardia* and *C. parvum* might explain why the co-existence of both pathogens in a single animal is only seen in exceptional cases. However, it should be kept in mind that the cited studies differ regarding the age of the animals and the method of parasite detection, e.g., immunofluorescence and/or PCR and, in the case of PCR, the chosen target genes. Considering that fecal sampling was performed only once in many studies and that the fecal shedding of *Giardia* often occurs sporadically and in low numbers, the prevalence of both parasites and coinfection might be underestimated [229]. Although both parasites can be present in healthy, asymptomatic animals [229,235], *Giardia* and *Cryptosporidium* are often associated with diarrhoea in young ruminants [220,231,234,246]. Unfortunately, most studies do not provide information about the severity of clinical signs animals infected with single or coinfections. Thus, it is difficult to say whether there is a relation between clinical outcome and the presence of *Giardia*-*Cryptosporidium* coinfection or not. However, in a study examining diarrhoea in 20 calves from birth until 4 months of age, an association between giardiasis and diarrhoea was shown. *Giardia* cysts were first detected at a mean age of 31.5 days, while *Cryptosporidium* oocysts were identified at a mean age of 16.3 days. All the calves were infected with both pathogens at some point during the study. *Cryptosporidium parvum* infection was cleared within 2 weeks in these animals, whereas *G. duodenalis* infection became chronic. The fact that these calves were diagnosed as *Cryptosporidium*-positive at an earlier time point in that study hints at the possibility that previous infection by *C. parvum* may promote later infection with *Giardia* [50,220].

#### 4.1.2. Eimeria

*Eimeria*, an Apicomplexa protozoan, is distributed all over the world and features high prevalence in ruminant livestock depending on the geographic area and host species. The parasite reproduces through a monoxenous life cycle and oocysts are excreted directly in the feces, with a patency of several days that may continue 2 weeks or more in calves [247,248]. *Eimeria* spp. are host-specific [249] and in cattle, 12 *Eimeria* species are assumed to be valid; the status of a further eight or more species is uncertain [248]. Fifteen *Eimeria* species in sheep and sixteen *Eimeria* species in goats have been described worldwide [250]. Only a few of these are considered to be pathogenic. In cattle, *E. bovis* and *E. zuernii* are the most important species and commonly associated with clinical coccidiosis, particularly in indoor housing systems [248,251,252,253], while *E. alabamensis* is identified as the predominant species on pasture [254]. *E. ovinoidalis* and *E. crandallis* are described as the most pathogenic species in sheep [255,256,257], whereas *E. ninakohlyakimovae*, *E. arloingi* and *E. caprina* regularly cause clinical coccidiosis in goats [258,259]. Depending on the *Eimeria* species as well as the host age and species, clinical coccidiosis results in moderate-to-severe diarrhoea with feces containing blood, fibrin and intestinal tissue, abdominal pain, fever, loss of appetite, weakness, dehydration, weight loss or even death [247,260]. The highest prevalence of *Eimeria* sp. shedding in cattle occurs in calves from 3 weeks up to 6 months of age [53,247,253] and clinical disease is mostly seen in young naïve animals, while adult animals are usually protected by immunity [247,258,260,261]. However, even if pathogenic species are present in farms and herds, coccidiosis might be subclinical [257,262]. Susceptibility to clinical disease is not only dependent on the *Eimeria* sp. involved, but possibly also due to infection pressure, virulence and the parasite’s replication potential. Immune response and the corresponding tissue inflammation, as well as additional factors such as stress, inadequate feeding and concurrent infections with other enteropathogens contribute to the severity of eimeriosis [248].

Frequently, mixed infections of two or more *Eimeria* species in individual animals have been reported [252,253,255,258,263,264,265], and it has been suggested that the severity of infection with a certain pathogenic *Eimeria* species may be aggravated by the presence of another, even if pathogenic in mono-infection; therefore, these interactions might be advantageous for some species [247,264]. Many studies demonstrated a high prevalence of *Eimeria* infections in ruminants [251,261,263,266] and techniques such as McMaster flotation were commonly used for diagnosis and parasite quantification. Concomitant viral, bacterial and protozoan pathogens were not considered in most studies on ruminant coccidiosis. Only a small number of investigations included the evaluation of coinfection by *Cryptosporidium* spp. [54,163,164,267,268] and a prevalence of coinfection ranging from 4 up to 10% was reported. These studies differed in methodology and species differentiation of *Cryptosporidium* and *Eimeria* was not performed in most of them. The prevalence of both *Eimeria* spp. and *Cryptosporidium* spp. is age-related [7,8,269]. Since *Eimeria* infection generally occurs in calves older than 3 weeks and *C. parvum* typically affects calves in the first two-to-three weeks of their life, simultaneous infection seems not to be a very common scenario and appears to be, most probable in 4 week-old calves if it occurrs at all [268]. Coinfection was also reported in 3–12 month-old calves in Estonia [267]; however, only a small number of samples was genotyped, demonstrating that cryptosporidia oocyst excretion was partly due to *C. andersoni*. A clinical relationship between *Eimeria* infection and cryptosporidiosis could not be shown in this study. Gulliksen et al. (2009) reported that in calves, concurrent infection with both pathogens was found more frequently in diarrheic feces than in normal samples. Unfortunately, in that study, no information about species identification was provided [163]. Currently, it can only be assumed that the exposure of calves to both pathogens, *Eimeria* and *Cryptosporidium*, may influence the clinical outcome and, in the case of infection by *C. parvum*, enteritis due to *C. parvum* at an early age may leave these calves more susceptible to clinical eimeriosis afterwards. However, no data are available to evidence this assumption. Considering that both parasites, *Cryptosporidium* and *Eimeria*, are highly prevalent in ruminant livestock, clinical disease caused by (sequential) exposure to both pathogens may be overlooked in field situations and epidemiological surveys.

### 4.2. Helminths

Depending on the geographical area and the kind of animal husbandry (indoor versus outdoor housing), infection by gastrointestinal helminths is a common threat for young ruminants all over the world. Diarrhoea is a typical clinical implication associated with nematode infection, especially in young animals, such as lambs [270]. *Strongyloides papillosus*, or species of the genera *Cooperia*, *Chabertia*, *Ostertagia*, *Haemonchus*, *Trichostrongylus*, *Buenostomum*, *Teladorsagia*, *Nematodirus* and *Trichuris* are regularly diagnosed in calves, goat kids and lambs [271,272,273,274,275,276]. Quite often, more than one type of nematode settles in the abomasum or intestines at the same time; it has been stated that mixed infections result in a greater impact on lamb productivity than single infections [270].

To date, only a small number of studies on gastrointestinal coinfections by protozoan parasites and helminths in ruminants has been published. The prevalence rates of mixed infection in a range from 1.7 to 38.3% in calves and lambs were reported [268,270,277]. Due to the shared resources and location within the host’s gastrointestinal tract, as well as the opposing immune responses protozoan parasites and helminths induce, coinfection with both could result in either facilitation or competition, and it has been assumed that the severity of disease may increase in coinfected animals [278]. Lambs at the age of 2 to 5 months were examined for infection with *Cryptosporidium*, *Giardia* and strongylid nematodes and it was found that coinfections exerted a greater impact on fecal consistency and body condition score than single infections [270]. Until now, investigations considering the interaction between protozoa and helminths have been performed almost exclusively in non-ruminants, showing effects on, for example, egg shedding and parasite abundance [279,280]. Experimental infection in mice demonstrated a higher worm burden and an increased duration of helminth egg shedding in the case of coinfection with coccidia or bacterial pathogens compared to singly infected mice, suggesting the reduced ability of coinfected mice to expel adult worms from the gut [280,281]. This may also apply to ruminants; however, to the best of our current knowledge, relevant studies are currently lacking. Additionally, lower peak oocyst shedding during a secondary coccidia challenge was reported in coinfected mice, leading to the hypothesis that tissue damage caused by helminth infection mimicked the ‘crowding effect’ of *Eimeria* infection [280]. Coinfection with helminths and protozoa seem to be highly common in nature [282]. However, whether early exposure to *C. parvum* or infection by other *Cryptosporidium* species later on favour simultaneous or subsequent nematode infection deserves further investigation. No information has been found in previous research on coinfection in young ruminants by *Cryptosporidium* and trematodes or cestodes; however, more investigations are needed to confirm this finding.

## 5. Conclusions

Many studies have been conducted on young diarrhoeic ruminants to identify causative pathogens and in many cases, coinfection with several agents has been detected [28,45,47,49,50,53,54,79,164]. However, most epidemiological studies are limited to particular infectious agents, resulting in an incomplete understanding of the epidemiological situations and ignoring the contribution other pathogens may make in coinfections [53]. Not only is there a gap in knowledge from a scientific point of view as a consequence, but there is also a difficulty in deducing optimal treatment and control recommendations for livestock exposed not only to *C. parvum*. Additionally, the effect of antiparasitic compounds against *C. parvum* may be affected by the presence of other enteropathogens, which is often not taken into account in efficacy studies [216]. The impact of coinfection on the severity of diarrhoeic disease has been suggested [30,49,61,283,284,285] and it has been assumed that the cumulative effects of concurrent pathogens infecting different parts of the alimentary tract may cause the loss of digestive and absorptive functions, resulting in diarrhoea [215]. However, experimental data on the impact of coinfection by *C. parvum* and other enteropathogens in ruminants are scarce. The simultaneous occurrence of different pathogens, including *C. parvum*, may facilitate or antagonise concurrent infections or may exert no respective effect [286]. A better understanding of these aspects would help to improve basic knowledge on host–pathogen interaction and the control of neonatal diarrhoea associated with cryptosporidiosis. This requires extensive research efforts that would not only contribute to improving sustained animal health and welfare but would also be of relevance in terms of the WHO’s “one health” concept, considering that *C. parvum* is a ubiquitously distributed zoonotic pathogen.

## References

[1] Sweeny J.P.A., Ryan U.M., Robertson I.D., Jacobson C. (2012). Prevalence and on-farm risk factors for diarrhoea in meat lamb flocks in Western Australia. Vet. J..

[2] Foster D.M., Smith G.W. (2009). Pathophysiology of diarrhea in calves. Vet. Clin. N. Am. Food Anim. Pract..

[3] Cheng Y., Yang C., Tan Z., He Z. (2021). Changes of Intestinal Oxidative Stress, Inflammation, and Gene Expression in Neonatal Diarrhoea Kids. Front. Vet. Sci..

[4] Tyzzer E.E. (1907). A sporozoan found in the peptic glands of the common mouse. Exp. Biol. Med..

[5] Cavalier-Smith T. (2014). Gregarine site-heterogeneous 18S rDNA trees, revision of gregarine higher classification, and the evolutionary diversification of Sporozoa. Eur. J. Protistol..

[6] Ryan U., Paparini A., Monis P., Hijjawi N. (2016). It’s official*—Cryptosporidium* is a gregarine. What are the implications for the water industry?. Water Res..

[7] Lendner M., Etzold M., Daugschies A. (2011). Kryptosporidiose—Ein Update. Berl. Und Munch. Tierarztl. Wochenschr..

[8] Santín M., Trout J.M., Xiao L., Zhou L., Greiner E., Fayer R. (2004). Prevalence and age-related variation of *Cryptosporidium* species and genotypes in dairy calves. Vet. Parasitol..

[9] Qi M., Zhang K., Huang M., Wang S., Xu C., Wang T., Jing B., Li J. (2020). Longitudinal detection of *Cryptosporidium* spp. in 1-10-week-old dairy calves on a farm in Xinjiang, China. Parasitol. Res..

[10] Fayer R., Santín M., Trout J.M., Greiner E. (2006). Prevalence of species and genotypes of *Cryptosporidium* found in 1-2-year-old dairy cattle in the eastern United States. Vet. Parasitol..

[11] Fan Y., Wang T., Koehler A.V., Hu M., Gasser R.B. (2017). Molecular investigation of *Cryptosporidium* and Giardia in pre- and post-weaned calves in Hubei Province, China. Parasites Vectors.

[12] Silverlås C., Näslund K., Björkman C., Mattsson J.G. (2010). Molecular characterisation of *Cryptosporidium* isolates from Swedish dairy cattle in relation to age, diarrhoea and region. Vet. Parasitol..

[13] Kváč M., Hromadová N., Květoňová D., Rost M., Sak B. (2011). Molecular characterization of *Cryptosporidium* spp. in pre-weaned dairy calves in the Czech Republic. Absence of *C. ryanae* and management-associated distribution of *C. andersoni*, *C. bovis* and *C. parvum* subtypes. Vet. Parasitol..

[14] Rieux A., Chartier C., Pors I., Paraud C. (2013). Dynamics of excretion and molecular characterization of *Cryptosporidium* isolates in pre-weaned French beef calves. Vet. Parasitol..

[15] Holzhausen I., Lendner M., Göhring F., Steinhöfel I., Daugschies A. (2019). Distribution of *Cryptosporidium parvum* gp60 subtypes in calf herds of Saxony, Germany. Parasitol. Res..

[16] Koinari M., Lymbery A.J., Ryan U.M. (2014). *Cryptosporidium* species in sheep and goats from Papua New Guinea. Exp. Parasitol..

[17] Tzanidakis N., Sotiraki S., Claerebout E., Ehsan A., Voutzourakis N., Kostopoulou D., Stijn C., Vercruysse J., Geurden T. (2014). Occurrence and molecular characterization of *Giardia duodenalis* and *Cryptosporidium* spp. in sheep and goats reared under dairy husbandry systems in Greece. Parasite.

[18] Kaupke A., Michalski M.M., Rzeżutka A. (2017). Diversity of *Cryptosporidium* species occurring in sheep and goat breeds reared in Poland. Parasitol. Res..

[19] Baroudi D., Hakem A., Adamu H., Amer S., Khelef D., Adjou K., Dahmani H., Chen X., Roellig D., Feng Y. (2018). Zoonotic *Cryptosporidium* species and subtypes in lambs and goat kids in Algeria. Parasites Vectors.

[20] Díaz P., Navarro E., Prieto A., Pérez-Creo A., Viña M., Díaz-Cao J.M., López C.M., Panadero R., Fernández G., Díez-Baños P. (2018). *Cryptosporidium* species in post-weaned and adult sheep and goats from N.W. Spain. Public and animal health significance. Vet. Parasitol..

[21] Quílez J., Torres E., Chalmers R.M., Hadfield S.J., del Cacho E., Sánchez-Acedo C. (2008). *Cryptosporidium* genotypes and subtypes in lambs and goat kids in Spain. Appl. Environ. Microbiol..

[22] Imre K., Luca C., Costache M., Sala C., Morar A., Morariu S., Ilie M.S., Imre M., Dărăbuş G. (2013). Zoonotic *Cryptosporidium parvum* in Romanian newborn lambs (Ovis aries). Vet. Parasitol..

[23] Díaz P., Quílez J., Prieto A., Navarro E., Pérez-Creo A., Fernández G., Panadero R., López C., Díez-Baños P., Morrondo P. (2015). *Cryptosporidium* species and subtype analysis in diarrhoeic pre-weaned lambs and goat kids from north-western Spain. Parasitol. Res..

[24] Papanikolopoulou V., Baroudi D., Guo Y., Wang Y., Papadopoulos E., Lafi S.Q., Abd El-Tawab M.M., Diakou A., Giadinis N.D., Feng Y. (2018). Genotypes and subtypes of *Cryptosporidium* spp. in diarrheic lambs and goat kids in northern Greece. Parasitol. Int..

[25] Dessì G., Tamponi C., Varcasia A., Sanna G., Pipia A.P., Carta S., Salis F., Díaz P., Scala A. (2020). *Cryptosporidium* infections in sheep farms from Italy. Parasitol. Res..

[26] Rivero-Juárez A., Dashti A., Santín M., Köster P.C., López-López P., Risalde M.A., García-Bocanegra I., Gómez-Villamandos J.C., Caballero-Gómez J., Frías M. (2021). Diarrhoea-causing enteric protist species in intensively and extensively raised pigs (Sus scrofa domesticus) in Southern Spain. Part II: Association with Hepatitis E virus susceptibility. Transbound. Emerg. Dis..

[27] Izzo M.M., Kirkland P.D., Mohler V.L., Perkins N.R., Gunn A.A., House J.K. (2011). Prevalence of major enteric pathogens in Australian dairy calves with diarrhoea. Aust. Vet. J..

[28] Cho Y., Han J., Wang C., Cooper V., Schwartz K., Engelken T., Yoon K. (2013). Case-control study of microbiological etiology associated with calf diarrhea. Vet. Microbiol..

[29] Singh S., Singh R., Singh K.P., Singh V., Malik Y.P.S., Kamdi B., Singh R., Kashyap G. (2020). Immunohistochemical and molecular detection of natural cases of bovine rotavirus and coronavirus infection causing enteritis in dairy calves. Microb. Pathog..

[30] Gomez D.E., Weese J.S. (2017). Viral enteritis in calves. Can. Vet. J. La Rev. Vet. Can..

[31] Torres-Medina A., Schlafer D.H., Mebus C.A. (1985). Rotaviral and coronaviral diarrhea. Vet. Clin. N. Am. Food Anim. Pract..

[32] Ramig R.F. (2004). Pathogenesis of intestinal and systemic rotavirus infection. J. Virol..

[33] Swiatek D.L., Palombo E.A., Lee A., Coventry M.J., Britz M.L., Kirkwood C.D. (2010). Detection and analysis of bovine rotavirus strains circulating in Australian calves during 2004 and 2005. Vet. Microbiol..

[34] Mebus C.A., Underdahl N.R., Rhodes M.B., Twiehaus M.J. Further studies on neonatal calf diarrhea virus. Proceedings of the Annual Meeting of the United States Animal Health Association.

[35] Flewett T.H., Bryden A.S., Davies H., Woode G.N., Bridger J.C., Derrick J.M. (1974). Relation between viruses from acute gastroenteritis of children and newborn calves. Lancet.

[36] Mihalov-Kovács E., Gellért Á., Marton S., Farkas S.L., Fehér E., Oldal M., Jakab F., Martella V., Bányai K. (2015). Candidate new rotavirus species in sheltered dogs, Hungary. Emerg. Infect. Dis..

[37] Bányai K., Kemenesi G., Budinski I., Földes F., Zana B., Marton S., Varga-Kugler R., Oldal M., Kurucz K., Jakab F. (2017). Candidate new rotavirus species in Schreiber’s bats, Serbia. Infect. Genet. Evol. J. Mol. Epidemiol. Evol. Genet. Infect. Dis..

[38] Timurkan M.Ö., Alkan F. (2020). Identification of rotavirus A strains in small ruminants. First detection of G8P1 genotypes in sheep in Turkey. Arch. Virol..

[39] Pourasgari F., Kaplon J., Karimi-Naghlani S., Fremy C., Otarod V., Ambert-Balay K., Mirjalili A., Pothier P. (2016). The molecular epidemiology of bovine rotaviruses circulating in Iran. A two-year study. Arch. Virol..

[40] Galindo-Cardiel I., Fernández-Jiménez M., Luján L., Buesa J., Espada J., Fantova E., Blanco J., Segalés J., Badiola J.J. (2011). Novel group A rotavirus G8 P1 as primary cause of an ovine diarrheic syndrome outbreak in weaned lambs. Vet. Microbiol..

[41] Papp H., Malik Y.S., Farkas S.L., Jakab F., Martella V., Bányai K. (2014). Rotavirus strains in neglected animal species including lambs, goats and camelids. Virusdisease.

[42] Chen F., Knutson T.P., Ciarlet M., Sturos M., Marthaler D.G. (2018). Complete genome characterization of a rotavirus B (RVB) strain identified in Alpine goat kids with enteritis reveals inter-species transmission with RVB bovine strains. J. Gen. Virol..

[43] Badaracco A., Garaicoechea L., Rodríguez D., Uriarte E.L., Odeón A., Bilbao G., Galarza R., Abdala A., Fernandez F., Parreño V. (2012). Bovine rotavirus strains circulating in beef and dairy herds in Argentina from 2004 to 2010. Vet. Microbiol..

[44] Chinsangaram J., Schore C.E., Guterbock W., Weaver L.D., Osburn B.I. (1995). Prevalence of group A and group B rotaviruses in the feces of neonatal dairy calves from California. Comp. Immunol. Microbiol. Infect. Dis..

[45] García A., Ruiz-Santa-Quiteria J.A., Orden J.A., Cid D., Sanz R., Gómez-Bautista M., de La Fuente R. (2000). Rotavirus and concurrent infections with other enteropathogens in neonatal diarrheic dairy calves in Spain. Comp. Immunol. Microbiol. Infect. Dis..

[46] Blanchard P.C. (2012). Diagnostics of dairy and beef cattle diarrhea. Vet. Clin. N. Am. Food Anim. Pract..

[47] Lanz Uhde F., Kaufmann T., Sager H., Albini S., Zanoni R., Schelling E., Meylan M. (2008). Prevalence of four enteropathogens in the faeces of young diarrhoeic dairy calves in Switzerland. Vet. Rec..

[48] De La Fuente R., García A., Ruiz-Santa-Quiteria J.A., Luzón M., Cid D., García S., Orden J.A., Gómez-Bautista M. (1998). Proportional morbidity rates of enteropathogens among diarrheic dairy calves in central Spain. Prev. Vet. Med..

[49] Göhring F., Möller-Holtkamp P., Daugschies A., Lendner M. (2014). Co-infections with *Cryptosporidium parvum* and other enteropathogenes support the occurrence and severity of diarrhoea in suckling calves. Tierarztl. Umsch..

[50] Gillhuber J., Rügamer D., Pfister K., Scheuerle M.C. (2014). Giardiosis and other enteropathogenic infections. A study on diarrhoeic calves in Southern Germany. BMC Res. Notes.

[51] Al Mawly J., Grinberg A., Prattley D., Moffat J., Marshall J., French N. (2015). Risk factors for neonatal calf diarrhoea and enteropathogen shedding in New Zealand dairy farms. Vet. J..

[52] Tzipori S., Sherwood D., Angus K.W., Campbell I., Gordon M. (1981). Diarrhea in lambs. Experimental infections with enterotoxigenic *Escherichia coli*, rotavirus, and *Cryptosporidium* sp.. Infect. Immun..

[53] Cruvinel L.B., Ayres H., Zapa D.M.B., Nicaretta J.E., Couto L.F.M., Heller L.M., Bastos T.S.A., Cruz B.C., Soares V.E., Teixeira W.F. (2020). Prevalence and risk factors for agents causing diarrhea (Coronavirus, Rotavirus, *Cryptosporidium* spp., Eimeria spp., and nematodes helminthes) according to age in dairy calves from Brazil. Trop. Anim. Health Prod..

[54] Lee S.H., Kim H.Y., Choi E.W., Kim D. (2019). Causative agents and epidemiology of diarrhea in Korean native calves. J. Vet. Sci..

[55] Lotfollahzadeh S., Madadgar O., Reza Mohebbi M., Reza Mokhber Dezfouli M., George Watson D. (2020). Bovine coronavirus in neonatal calf diarrhoea in Iran. Vet. Med. Sci..

[56] Stair E.L., Rhodes M.B., White R.G., Mebus C.A. (1972). Neonatal calf diarrhea. Purification and electron microscopy of a coronavirus-like agent. Am. J. Vet. Res..

[57] Mebus C.A., White R.G., Stair E.L., Rhodes M.B., Twiehaus M.J. (1972). Neonatal calf diarrhea. Results of a field trial using a reo-like virus vaccine. Vet. Med. Small Anim. Clin..

[58] Mebus C.A., Stair E.L., Rhodes M.B., Twiehaus M.J. (1973). Neonatal calf diarrhea. Propagation, attenuation, and characteristics of a coronavirus-like agent. Am. J. Vet. Res..

[59] Clark M.A. (1993). Bovine coronavirus. Br. Vet. J..

[60] Fulton R.W., Herd H.R., Sorensen N.J., Confer A.W., Ritchey J.W., Ridpath J.F., Burge L.J. (2015). Enteric disease in postweaned beef calves associated with Bovine coronavirus clade 2. J. Vet. Diagn. Investig..

[61] Boileau M.J., Kapil S. (2010). Bovine coronavirus associated syndromes. Vet. Clin. N. Am. Food Anim. Pract..

[62] Bartels C.J.M., Holzhauer M., Jorritsma R., Swart W.A.J.M., Lam T.J.G.M. (2010). Prevalence, prediction and risk factors of enteropathogens in normal and non-normal faeces of young Dutch dairy calves. Prev. Vet. Med..

[63] Lora I., Gottardo F., Contiero B., Dall Ava B., Bonfanti L., Stefani A., Barberio A. (2018). Association between passive immunity and health status of dairy calves under 30 days of age. Prev. Vet. Med..

[64] Haschek B., Klein D., Benetka V., Herrera C., Sommerfeld-Stur I., Vilcek S., Moestl K., Baumgartner W. (2006). Detection of bovine torovirus in neonatal calf diarrhoea in Lower Austria and Styria (Austria). J. Vet. Med. B Infect. Dis. Vet. Public Health.

[65] Gomez D.E., Arroyo L.G., Poljak Z., Viel L., Weese J.S. (2017). Detection of Bovine Coronavirus in Healthy and Diarrheic Dairy Calves. J. Vet. Intern. Med..

[66] Alfieri A.A., Ribeiro J., de Carvalho Balbo L., Lorenzetti E., Alfieri A.F. (2018). Dairy calf rearing unit and infectious diseases. Diarrhea outbreak by bovine coronavirus as a model for the dispersion of pathogenic microorganisms. Trop. Anim. Health Prod..

[67] Muñoz M., Alvarez M., Lanza I., Cármenes P. (1996). Role of enteric pathogens in the aetiology of neonatal diarrhoea in lambs and goat kids in Spain. Epidemiol. Infect..

[68] Eisa M.I., Mohamed A. (2004). Role of enteric pathogens in enteritis in lambs, goat kids and children and their zoonotic importance. Vet. Med. J. Giza.

[69] Ozmen O., Yukari B.A., Haligur M., Sahinduran S. (2006). Observations and immunohistochemical detection of Coronavirus, *Cryptosporidium parvum* and Giardia intestinalis in neonatal diarrhoea in lambs and kids. Schweiz. Arch. Tierheilkd..

[70] Tråvén M., Carlsson U., Lundén A., Larsson B. (1999). Serum antibodies to bovine coronavirus in Swedish sheep. Acta Vet. Vet. Scand..

[71] Burimuah V., Sylverken A., Owusu M., El-Duah P., Yeboah R., Lamptey J., Frimpong Y.O., Agbenyega O., Folitse R., Tasiame W. (2020). Sero-prevalence, cross-species infection and serological determinants of prevalence of Bovine Coronavirus in Cattle, Sheep and Goats in Ghana. Vet. Microbiol..

[72] Gumusova O., Yazici Z., Albayrak H., Çakiroglu D. (2007). First report of bovine rotavirus and bovine coronavirus seroprevalance in goats in Turkey. Vet Glas.

[73] Chae J., Park J., Jung S., Kang J., Chae J., Choi K. (2019). Acute phase response in bovine coronavirus positive post-weaned calves with diarrhea. Acta Vet. Scand..

[74] Amer H.M., Abd El Wahed A., Shalaby M.A., Almajhdi F.N., Hufert F.T., Weidmann M. (2013). A new approach for diagnosis of bovine coronavirus using a reverse transcription recombinase polymerase amplification assay. J. Virol. Methods.

[75] Lojkić I., Krešić N., Šimić I., Bedeković T. (2015). Detection and molecular characterisation of bovine corona and toroviruses from Croatian cattle. BMC Vet. Res..

[76] Bok M., Alassia M., Frank F., Vega C.G., Wigdorovitz A., Parreño V. (2018). Passive immunity to control Bovine coronavirus diarrhea in a dairy herd in Argentina. Rev. Argent. Microbiol..

[77] Kapil S., Trent A.M., Goyal S.M. (1990). Excretion and persistence of bovine coronavirus in neonatal calves. Arch. Virol..

[78] Oma V.S., Tråvén M., Alenius S., Myrmel M., Stokstad M. (2016). Bovine coronavirus in naturally and experimentally exposed calves; viral shedding and the potential for transmission. Virol. J..

[79] de La Fuente R., Luzón M., Ruiz-Santa-Quiteria J.A., García A., Cid D., Orden J.A., García S., Sanz R., Gómez-Bautista M. (1999). *Cryptosporidium* and concurrent infections with other major enterophatogens in 1 to 30-day-old diarrheic dairy calves in central Spain. Vet. Parasitol..

[80] Alfred N., Liu H., Li M.L., Hong S.F., Tang H.B., Wei Z.Z., Chen Y., Li F.K., Zhong Y.Z., Huang W.J. (2015). Molecular epidemiology and phylogenetic analysis of diverse bovine astroviruses associated with diarrhea in cattle and water buffalo calves in China. J. Vet. Med. Sci..

[81] Woode G.N., Bridger J.C. (1978). Isolation of small viruses resembling astroviruses and caliciviruses from acute enteritis of calves. J. Med. Microbiol..

[82] Woode G.N., Pohlenz J.F., Gourley N.E., Fagerland J.A. (1984). Astrovirus and Breda virus infections of dome cell epithelium of bovine ileum. J. Clin. Microbiol..

[83] Snodgrass D.R., Gray E.W. (1977). Detection and transmission of 30 nm virus particles (astroviruses) in faeces of lambs with diarrhoea. Arch. Virol..

[84] Snodgrass D.R., Angus K.W., Gray E.W., Menzies J.D., Paul G. (1979). Pathogenesis of diarrhoea caused by astrovirus infections in lambs. Arch. Virol..

[85] Sharp C.P., Gregory W.F., Mason C., Barend M., Beard P.M. (2015). High prevalence and diversity of bovine astroviruses in the faeces of healthy and diarrhoeic calves in South West Scotland. Vet. Microbiol..

[86] Mohamed F.F., Mansour S.M.G., El-Araby I.E., Mor S.K., Goyal S.M. (2017). Molecular detection of enteric viruses from diarrheic calves in Egypt. Arch. Virol..

[87] Yamashita T., Ito M., Kabashima Y., Tsuzuki H., Fujiura A., Sakae K. (2003). Isolation and characterization of a new species of kobuvirus associated with cattle. J. Gen. Virol..

[88] Khamrin P., Maneekarn N., Peerakome S., Okitsu S., Mizuguchi M., Ushijima H. (2008). Bovine kobuviruses from cattle with diarrhea. Emerg. Infect. Dis..

[89] Barry A.F., Ribeiro J., Alfieri A.F., van der Poel W.H.M., Alfieri A.A. (2011). First detection of kobuvirus in farm animals in Brazil and the Netherlands. Infect. Genet. Evol. J. Mol. Epidemiol. Evol. Genet. Infect. Dis..

[90] Jeoung H., Lim J., Jeong W., Oem J., An D. (2011). Three clusters of bovine kobuvirus isolated in Korea, 2008–2010. Virus Genes.

[91] Di Martino B., di Profio F., di Felice E., Ceci C., Pistilli M.G., Marsilio F. (2012). Molecular detection of bovine kobuviruses in Italy. Arch. Virol..

[92] Mohamed F.F., Mansour S.M.G., Orabi A., El-Araby I.E., Ng T.F.F., Mor S.K., Goyal S.M. (2018). Detection and genetic characterization of bovine kobuvirus from calves in Egypt. Arch. Virol..

[93] Wang L., Fredrickson R., Duncan M., Samuelson J., Hsiao S. (2020). Bovine Kobuvirus in Calves with Diarrhea, United States. Emerg. Infect. Dis..

[94] Işidan H., Turan T., Atasoy M.O., Sözdutmaz I., Irehan B. (2019). Detection and first molecular characterisation of three picornaviruses from diarrhoeic calves in Turkey. Acta Vet. Hung..

[95] Blas-Machado U., Saliki J.T., Sánchez S., Brown C.C., Zhang J., Keys D., Woolums A., Harvey S.B. (2011). Pathogenesis of a bovine enterovirus-1 isolate in experimentally infected calves. Vet. Pathol..

[96] Sobhy N.M., Mor S.K., Mohammed M.E.M., Bastawecy I.M., Fakhry H.M., Youssef C.R.B., Abouzeid N.Z., Goyal S.M. (2015). Isolation and molecular characterization of bovine enteroviruses in Egypt. Vet. J..

[97] Omatsu T., Tsuchiaka S., Hirata T., Shiroma Y., Okazaki S., Katayama Y., Oba M., Nishiura N., Sassa Y., Furuya T. (2014). Detection of enterovirus genome sequence from diarrheal feces of goat. Virus Genes.

[98] Di Martino B., di Profio F., Martella V., Ceci C., Marsilio F. (2011). Evidence for recombination in neboviruses. Vet. Microbiol..

[99] Günther H., Otto P., Heilmann P. (1984). Diarrhea in young calves. 6. Determination of the pathogenicity of a bovine coronavirus and an unidentified icosahedral virus. Arch. Exp. Veterinarmed..

[100] Günther H., Otto P. (1987). Diarrhea in young calves. 7. “Zackenvirus” (Jena agent 117/80)-a new diarrhea pathogen in calves. Arch. Exp. Veterinarmed..

[101] Otto P.H., Clarke I.N., Lambden P.R., Salim O., Reetz J., Liebler-Tenorio E.M. (2011). Infection of calves with bovine norovirus GIII.1 strain Jena virus. An experimental model to study the pathogenesis of norovirus infection. J. Virol..

[102] Jung K., Scheuer K.A., Zhang Z., Wang Q., Saif L.J. (2014). Pathogenesis of GIII.2 bovine norovirus, CV186-OH/00/US strain in gnotobiotic calves. Vet. Microbiol..

[103] Oliver S.L., Wood E., Asobayire E., Wathes D.C., Brickell J.S., Elschner M., Otto P., Lambden P.R., Clarke I.N., Bridger J.C. (2007). Serotype 1 and 2 bovine noroviruses are endemic in cattle in the United kingdom and Germany. J. Clin. Microbiol..

[104] Mauroy A., Scipioni A., Mathijs E., Saegerman C., Mast J., Bridger J.C., Ziant D., Thys C., Thiry E. (2009). Epidemiological study of bovine norovirus infection by RT-PCR and a VLP-based antibody ELISA. Vet. Microbiol..

[105] Thomas C., Jung K., Han M., Hoet A., Scheuer K., Wang Q., Saif L.J. (2014). Retrospective serosurveillance of bovine norovirus (GIII.2) and nebovirus in cattle from selected feedlots and a veal calf farm in 1999 to 2001 in the United States. Arch. Virol..

[106] van der Poel W.H.M., van der Heide R., Verschoor F., Gelderblom H., Vinjé J., Koopmans M.P.G. (2003). Epidemiology of Norwalk-like virus infections in cattle in The Netherlands. Vet. Microbiol..

[107] Jor E., Myrmel M., Jonassen C.M. (2010). SYBR Green based real-time RT-PCR assay for detection and genotype prediction of bovine noroviruses and assessment of clinical significance in Norway. J. Virol. Methods.

[108] Di Martino B., di Profio F., di Felice E., Melegari I., Ceci C., Mauroy A., Thiry E., Martella V., Marsilio F. (2014). Genetic heterogeneity of bovine noroviruses in Italy. Arch. Virol..

[109] Wolf S., Williamson W., Hewitt J., Lin S., Rivera-Aban M., Ball A., Scholes P., Savill M., Greening G.E. (2009). Molecular detection of norovirus in sheep and pigs in New Zealand farms. Vet. Microbiol..

[110] Carstens E.B. (2010). Ratification vote on taxonomic proposals to the International Committee on Taxonomy of Viruses (2009). Arch. Virol..

[111] Smiley J.R., Chang K.O., Hayes J., Vinjé J., Saif L.J. (2002). Characterization of an enteropathogenic bovine calicivirus representing a potentially new calicivirus genus. J. Virol..

[112] Oliver S.L., Asobayire E., Dastjerdi A.M., Bridger J.C. (2006). Genomic characterization of the unclassified bovine enteric virus Newbury agent-1 (Newbury1) endorses a new genus in the family Caliciviridae. Virology.

[113] Bridger J.C., Hall G.A., Brown J.F. (1984). Characterization of a calici-like virus (Newbury agent) found in association with astrovirus in bovine diarrhea. Infect. Immun..

[114] Hassine-Zaafrane M., Kaplon J., Sdiri-Loulizi K., Aouni Z., Pothier P., Aouni M., Ambert-Balay K. (2012). Molecular prevalence of bovine noroviruses and neboviruses detected in central-eastern Tunisia. Arch. Virol..

[115] Kaplon J., Guenau E., Asdrubal P., Pothier P., Ambert-Balay K. (2011). Possible novel nebovirus genotype in cattle, France. Emerg. Infect. Dis..

[116] Candido M., Alencar A.L.F., Almeida-Queiroz S.R., Buzinaro M.G., Munin F.S., Godoy S.H.S., Livonesi M.C., Fernandes A.M., Sousa R.L. (2016). First detection and molecular characterization of Nebovirus in Brazil. Epidemiol. Infect..

[117] Pourasgari F., Kaplon J., Sanchooli A., Fremy C., Karimi-Naghlani S., Otarod V., Ambert-Balay K., Mojgani N., Pothier P. (2018). Molecular prevalence of bovine noroviruses and neboviruses in newborn calves in Iran. Arch. Virol..

[118] Turan T., Işıdan H., Atasoy M.O., Irehan B. (2018). Detection and Molecular Analysis of Bovine Enteric Norovirus and Nebovirus in Turkey. J. Vet. Res..

[119] Guo Z., He Q., Zhang B., Yue H., Tang C. (2019). Detection and molecular characteristics of neboviruses in dairy cows in China. J. Gen. Virol..

[120] Karayel-Hacioglu I., Alkan F. (2019). Molecular characterization of bovine noroviruses and neboviruses in Turkey. Detection of recombinant strains. Arch. Virol..

[121] Woode G.N., Reed D.E., Runnels P.L., Herrig M.A., Hill H.T. (1982). Studies with an unclassified virus isolated from diarrheic calves. Vet. Microbiol..

[122] Hoet A.E., Nielsen P.R., Hasoksuz M., Thomas C., Wittum T.E., Saif L.J. (2003). Detection of bovine torovirus and other enteric pathogens in feces from diarrhea cases in cattle. J. Vet. Diagn. Investig..

[123] Jamieson F.B., Wang E.E., Bain C., Good J., Duckmanton L., Petric M. (1998). Human torovirus. A new nosocomial gastrointestinal pathogen. J. Infect. Dis..

[124] Hoet A.E., Saif L.J. (2004). *Bovine torovirus* (Breda virus) revisited. Anim. Health Res. Rev..

[125] Hoet A.E., Smiley J., Thomas C., Nielsen P.R., Wittum T.E., Saif L.J. (2003). Association of enteric shedding of bovine torovirus (Breda virus) and other enteropathogens with diarrhea in veal calves. Am. J. Vet. Res..

[126] Koopmans M., Cremers H., Woode G., Horzinek M.C. (1990). Breda virus (Toroviridae) infection and systemic antibody response in sentinel calves. Am. J. Vet. Res..

[127] Pérez E., Kummeling A., Janssen M.M., Jiménez C., Alvarado R., Caballero M., Donado P., Dwinger R.H. (1998). Infectious agents associated with diarrhoea of calves in the canton of Tilarán, Costa Rica. Prev. Vet. Med..

[128] Aita T., Kuwabara M., Murayama K., Sasagawa Y., Yabe S., Higuchi R., Tamura T., Miyazaki A., Tsunemitsu H. (2012). Characterization of epidemic diarrhea outbreaks associated with bovine torovirus in adult cows. Arch. Virol..

[129] Nogueira J.S., Asano K.M., de Souza S.P., Brandão P.E., Richtzenhain L.J. (2013). First detection and molecular diversity of Brazilian bovine torovirus (BToV) strains from young and adult cattle. Res. Vet. Sci..

[130] Hoet A.E., Cho K.O., Chang K.O., Loerch S.C., Wittum T.E., Saif L.J. (2002). Enteric and nasal shedding of bovine torovirus (Breda virus) in feedlot cattle. Am. J. Vet. Res..

[131] Ito T., Okada N., Okawa M., Fukuyama S., Shimizu M. (2009). Detection and characterization of bovine torovirus from the respiratory tract in Japanese cattle. Vet. Microbiol..

[132] van Kruiningen H.J., Castellano V.P., Koopmans M., Harris L.L. (1992). A serologic investigation for coronavirus and Breda virus antibody in winter dysentery of dairy cattle in the northeastern United States. J. Vet. Diagn. Investig..

[133] Duckmanton L., Carman S., Nagy E., Petric M. (1998). Detection of bovine torovirus in fecal specimens of calves with diarrhea from Ontario farms. J. Clin. Microbiol..

[134] Matiz K., Kecskeméti S., Kiss I., Adám Z., Tanyi J., Nagy B. (2002). Torovirus detection in faecal specimens of calves and pigs in Hungary. Short communication. Acta Vet. Hung..

[135] Gülaçtı I., Işıdan H., Sözdutmaz I. (2014). Detection of bovine torovirus in fecal specimens from calves with diarrhea in Turkey. Arch. Virol..

[136] Brown D.W., Beards G.M., Flewett T.H. (1987). Detection of Breda virus antigen and antibody in humans and animals by enzyme immunoassay. J. Clin. Microbiol..

[137] Koopmans M., van den Boom U., Woode G., Horzinek M.C. (1989). Seroepidemiology of Breda virus in cattle using ELISA. Vet. Microbiol..

[138] Liebler E.M., Klüver S., Pohlenz J., Koopmans M. (1992). Zur Bedeutung des Bredavirus als Durchfallerreger in niedersächsischen Kälberbeständen. DTW. Dtsch. Tierarztl. Wochenschr..

[139] Kuwabara M., Wada K., Maeda Y., Miyazaki A., Tsunemitsu H. (2007). First isolation of cytopathogenic bovine torovirus in cell culture from a calf with diarrhea. Clin. Vaccine Immunol..

[140] Escherich T. (1989). The intestinal bacteria of the neonate and breast-fed infant. Rev. Infect. Dis..

[141] Welch R.A., Dworkin M., Falkow S., Rosenberg E., Schleifer K.H., Stackebrandt E. (2006). The Genus Escherichia.

[142] Nataro J.P., Kaper J.B. (1998). Diarrheagenic *Escherichia coli*. Clin. Microbiol. Rev..

[143] Kaper J.B., Nataro J.P., Mobley H.L. (2004). Pathogenic *Escherichia coli*. Nat. Rev. Microbiol..

[144] Croxen M.A., Law R.J., Scholz R., Keeney K.M., Wlodarska M., Finlay B.B. (2013). Recent advances in understanding enteric pathogenic *Escherichia coli*. Clin. Microbiol. Rev..

[145] Janke B.H., Francis D.H., Collins J.E., Libal M.C., Zeman D.H., Johnson D.D. (1989). Attaching and effacing *Escherichia coli* infections in calves, pigs, lambs, and dogs. J. Vet. Diagn. Investig..

[146] Duhamel G.E., Moxley R.A., Maddox C.W., Erickson E.D. (1992). Enteric infection of a goat with enterohemorrhagic *Escherichia coli* (O103:H2). J. Vet. Diagn. Investig..

[147] Drolet R., Fairbrother J.M., Vaillancourt D. (1994). Attaching and effacing *Escherichia coli* in a goat with diarrhea. Can. Vet. J. La Rev. Vet. Can..

[148] Kang S.J., Ryu S.J., Chae J.S., Eo S.K., Woo G.J., Lee J.H. (2004). Occurrence and characteristics of enterohemorrhagic *Escherichia coli* O157 in calves associated with diarrhoea. Vet. Microbiol..

[149] Shahrani M., Dehkordi F.S., Momtaz H. (2014). Characterization of *Escherichia coli* virulence genes, pathotypes and antibiotic resistance properties in diarrheic calves in Iran. Biol. Res..

[150] Fakih I., Thiry D., Duprez J.-N., Saulmont M., Iguchi A., Piérard D., Jouant L., Daube G., Ogura Y., Hayashi T. (2017). Identification of Shiga toxin-producing (STEC) and enteropathogenic (EPEC) *Escherichia coli* in diarrhoeic calves and comparative genomics of O5 bovine and human STEC. Vet. Microbiol..

[151] Awad W.S., El-Sayed A.A., Mohammed F.F., Bakry N.M., Abdou N.M.I., Kamel M.S. (2020). Molecular characterization of pathogenic *Escherichia coli* isolated from diarrheic and in-contact cattle and buffalo calves. Trop. Anim. Health Prod..

[152] de la Fuente R., Garcia S., Orden J.A., Ruiz-Santa-Quiteria J.A., Diez R., Cid D. (2002). Prevalence and characteristics of attaching and effacing strains of *Escherichia coli* isolated from diarrheic and healthy sheep and goats. Am. J. Vet. Res..

[153] Orden J.A., Cid D., Ruiz-Santa-Quiteria J.A., García S., Martínez S., de La Fuente R. (2002). Verotoxin-producing *Escherichia coli* (VTEC), enteropathogenic E. coli (EPEC) and necrotoxigenic E. coli (NTEC) isolated from healthy cattle in Spain. J. Appl. Microbiol..

[154] Kolenda R., Burdukiewicz M., Schierack P. (2015). A systematic review and meta-analysis of the epidemiology of pathogenic *Escherichia coli* of calves and the role of calves as reservoirs for human pathogenic *E. coli*. Front. Cell. Infect. Microbiol..

[155] La Ragione R.M., Best A., Woodward M.J., Wales A.D. (2009). *Escherichia coli* O157:H7 colonization in small domestic ruminants. FEMS Microbiol. Rev..

[156] La Ragione R.M., Best A., Clifford D., Weyer U., Johnson L., Marshall R.N., Cooley W.A., Farrelly S., Pearson G.R., Woodward M.J. (2006). Influence of colostrum deprivation and concurrent *Cryptosporidium parvum* infection on the colonization and persistence of *Escherichia coli* O157. H7 in young lambs. J. Med. Microbiol..

[157] Orden J.A., Ruiz-Santa-Quiteria J.A., Cid D., García S., de La Fuente R. (1999). Prevalence and characteristics of necrotoxigenic *Escherichia coli* (NTEC) strains isolated from diarrhoeic dairy calves. Vet. Microbiol..

[158] van Bost S., Roels S., Mainil J. (2001). Necrotoxigenic *Escherichia coli* type-2 invade and cause diarrhoea during experimental infection in colostrum-restricted newborn calves. Vet. Microbiol..

[159] Blanco M., Blanco J., Blanco J.E., Ramos J. (1993). Enterotoxigenic, verotoxigenic, and necrotoxigenic *Escherichia coli* isolated from cattle in Spain. Am. J. Vet. Res..

[160] Burns A.L., Ball H.J., Finlay D.A. (1996). CNF producing *Escherichia coli* isolated from cattle in Northern Ireland. Vet. Microbiol..

[161] Osek J. (2001). Characterization of necrotoxigenic *Escherichia coli* (NTEC) strains isolated from healthy calves in Poland. J. Vet. Med. B Infect. Dis. Vet. Public Health.

[162] Naciri M., Paul Lefay M., Mancassola R., Poirier P., Chermette R. (1999). Role of *Cryptosporidium parvum* as a pathogen in neonatal diarrhoea complex in suckling and dairy calves in France. Vet. Parasitol..

[163] Gulliksen S.M., Jor E., Lie K.I., Hamnes I.S., Løken T., Akerstedt J., Osterås O. (2009). Enteropathogens and risk factors for diarrhea in Norwegian dairy calves. J. Dairy Sci..

[164] Brar A.P.S., Sood N.K., Kaur P., Singla L.D., Sandhu B.S., Gupta K., Narang D., Singh C.K., Chandra M. (2017). Periurban outbreaks of bovine calf scours in Northern India caused by *Cryptosporidium* in association with other enteropathogens. Epidemiol. Infect..

[165] Gouet P., Contrepois M., Dubourguier H.C., Riou Y., Scherrer R., Laporte J., Vautherot J.F., Cohen J., L’Haridon R. (1978). The experimental production of diarrhoea in colostrum deprived axenic and gnotoxenic calves with enteropathogenic *Escherichia coli*, rotavirus, coronavirus and in a combined infection of rotavirus and *E. coli*. Annales de recherches veterinaires. Ann. Vet. Res..

[166] Tzipori S.R., Makin T.J., Smith M.L., Krautil F.L. (1981). Clinical manifestations of diarrhea in calves infected with rotavirus and enterotoxigenic *Escherichia coli*. J. Clin. Microbiol..

[167] Snodgrass D.R., Smith M.L., Krautil F.L. (1982). Interaction of rotavirus and enterotoxigenic *Escherichia coli* in conventionally-reared dairy calves. Vet. Microbiol..

[168] Hess R.G., Bachmann P.A., Baljer G., Mayr A., Pospischil A., Schmid G. (1984). Synergism in experimental mixed infections of newborn colostrum-deprived calves with bovine rotavirus and enterotoxigenic *Escherichia coli* (ETEC). Zent. Veterinarmed. Reihe B J. Vet. Med. Ser. B.

[169] Runnels P.L., Moon H.W., Matthews P.J., Whipp S.C., Woode G.N. (1986). Effects of microbial and host variables on the interaction of rotavirus and *Escherichia coli* infections in gnotobiotic calves. Am. J. Vet. Res..

[170] Kiu R., Hall L.J. (2018). An update on the human and animal enteric pathogen Clostridium perfringens. Emerg. Microbes Infect..

[171] Vance H.N. (1967). Clostridium perfringens as a pathogen of cattle. A literature review. Can. J. Comp. Med. Vet. Sci..

[172] Songer J.G. (1996). Clostridial enteric diseases of domestic animals. Clin. Microbiol. Rev..

[173] Simpson K.M., Callan R.J., van Metre D.C. (2018). Clostridial Abomasitis and Enteritis in Ruminants. Vet. Clin. N. Am. Food Anim. Pract..

[174] Welch W.H., Nuttall G.H.F. (1892). A gas-producing bacillus (bacillus aerogenes capsulatas, nov. spec.) capable of rapid development in the blood vessels after death. John Hopkins Hosp. Bull..

[175] Rood J.I., Adams V., Lacey J., Lyras D., McClane B.A., Melville S.B., Moore R.J., Popoff M.R., Sarker M.R., Songer J.G. (2018). Expansion of the Clostridium perfringens toxin-based typing scheme. Anaerobe.

[176] Manteca C., Daube G., Pirson V., Limbourg B., Kaeckenbeeck A., Mainil J.G. (2001). Bacterial intestinal flora associated with enterotoxaemia in Belgian Blue calves. Vet. Microbiol..

[177] Dray T. (2004). Clostridium perfringens type A and beta2 toxin associated with enterotoxemia in a 5-week-old goat. Can. Vet. J. La Rev. Vet. Can..

[178] Miyakawa M.E.F., Saputo J., Leger J.S., Puschner B., Fisher D.J., McClane B.A., Uzal F.A. (2007). Necrotizing enterocolitis and death in a goat kid associated with enterotoxin (CPE)-producing Clostridium perfringens type A. Can. Vet. J. = La Rev. Vet. Can..

[179] van Kruiningen H.J., Nyaoke C.A., Sidor I.F., Fabis J.J., Hinckley L.S., Lindell K.A. (2009). Clostridial abomasal disease in Connecticut dairy calves. Can. Vet. J. = La Rev. Vet. Can..

[180] Savic B., Prodanovic R., Ivetic V., Radanovic O., Bojkovski J. (2012). Enteritis associated with Clostridium perfringens type A in 9-month-old calves. Can. Vet. J. La Rev. Vet. Can..

[181] Selim A.M., Elhaig M.M., Zakaria I., Ali A. (2017). Bacteriological and molecular studies of Clostridium perfringens infections in newly born calves. Trop. Anim. Health Prod..

[182] Gkiourtzidis K., Frey J., Bourtzi-Hatzopoulou E., Iliadis N., Sarris K. (2001). PCR detection and prevalence of alpha-, beta-, beta 2-, epsilon-, iota- and enterotoxin genes in Clostridium perfringens isolated from lambs with clostridial dysentery. Vet. Microbiol..

[183] Munday J.S., Bentall H., Aberdein D., Navarro M., Uzal F.A., Brown S. (2020). Death of a neonatal lamb due to Clostridium perfringens type B in New Zealand. N. Zeal. Vet. J..

[184] Niilo L., Harries W.N., Jones G.A. (1974). Clostridium perfringens type C in hemorrhagic enterotoxemia of neonatal calves in Alberta. Can. Vet. J. = La Rev. Vet. Can..

[185] Uzal F.A., Songer J. (2008). Glenn Diagnosis of Clostridium perfringens intestinal infections in sheep and goats. J. Vet. Diagn. Investig. Off. Publ. Am. Assoc. Vet. Lab. Diagn. Inc..

[186] Garcia J.P., Anderson M., Blanchard P., Mete A., Uzal F.A. (2013). The pathology of enterotoxemia by Clostridium perfringens type C in calves. J. Vet. Diagn. Investig..

[187] Uzal F.A., Kelly W.R. (1998). Experimental Clostridium perfringens type D enterotoxemia in goats. Vet. Pathol..

[188] Singh D.D., Pawaiya R.S., Gururaj K., Gangwar N.K., Mishra A.K., Andani D., Singh M.K., Bhushan S., Kumar A. (2018). Molecular detection of Clostridium perfringens toxinotypes, Enteropathogenic *Escherichia coli*, rotavirus and coronavirus in diarrheic fecal samples of neonatal goat kids. Vet. Arhiv..

[189] Billington S.J., Wieckowski E.U., Sarker M.R., Bueschel D., Songer J.G., McClane B.A. (1998). *Clostridium perfringens* type E animal enteritis isolates with highly conserved, silent enterotoxin gene sequences. Infect. Immun..

[190] Songer J.G., Miskimmins D.W. (2004). Clostridium perfringens type E enteritis in calves. Two cases and a brief review of the literature. Anaerobe.

[191] Kim H., Byun J., Roh I., Bae Y., Lee M., Kim B., Songer J.G., Jung B.Y. (2013). First isolation of Clostridium perfringens type E from a goat with diarrhea. Anaerobe.

[192] Rodriguez-Palacios A., Stämpfli H.R., Duffield T., Peregrine A.S., Trotz-Williams L.A., Arroyo L.G., Brazier J.S., Weese J.S. (2006). Clostridium difficile PCR ribotypes in calves, Canada. Emerg. Infect. Dis..

[193] Rodriguez-Palacios A., Stämpfli H.R., Stalker M., Duffield T., Weese J.S. (2007). Natural and experimental infection of neonatal calves with *Clostridium difficile*. Vet. Microbiol..

[194] Hammitt M.C., Bueschel D.M., Keel M.K., Glock R.D., Cuneo P., DeYoung D.W., Reggiardo C., Trinh H.T., Songer J.G. (2008). A possible role for *Clostridium difficile* in the etiology of calf enteritis. Vet. Microbiol..

[195] Schneeberg A., Neubauer H., Schmoock G., Grossmann E., Seyboldt C. (2013). Presence of Clostridium difficile PCR ribotype clusters related to 033, 078 and 045 in diarrhoeic calves in Germany. J. Med. Microbiol..

[196] Magistrali C.F., Maresca C., Cucco L., Bano L., Drigo I., Filippini G., Dettori A., Broccatelli S., Pezzotti G. (2015). Prevalence and risk factors associated with Clostridium difficile shedding in veal calves in Italy. Anaerobe.

[197] Eng S., Pusparajah P., Ab Mutalib N., Ser H., Chan K., Lee L.S. (2015). A review on pathogenesis, epidemiology and antibiotic resistance. Front. Life Sci..

[198] Holschbach C.L., Peek S.F. (2018). Salmonella in Dairy Cattle. Vet. Clin. N. Am. Food Anim. Pract..

[199] Barrington G.M., Gay J.M., Evermann J.F. (2002). Biosecurity for neonatal gastrointestinal diseases. Vet. Clin. N. Am. Food Anim. Pract..

[200] Habing G.G., Neuder L.M., Raphael W., Piper-Youngs H., Kaneene J.B. (2011). Efficacy of oral administration of a modified-live Salmonella Dublin vaccine in calves. J. Am. Vet. Med. Assoc..

[201] Wray C., Sojka W.J. (1978). Experimental Salmonella typhimurium infection in calves. Res. Vet. Sci..

[202] Wray C., Sojka W.J. (1981). Salmonella dublin infection of calves. Use of small doses to simulate natural infection on the farm. J. Hyg..

[203] Fossler C.P., Wells S.J., Kaneene J.B., Ruegg P.L., Warnick L.D., Bender J.B., Eberly L.E., Godden S.M., Halbert L.W. (2005). Herd-level factors associated with isolation of Salmonella in a multi-state study of conventional and organic dairy farms II. Salmonella shedding in calves. Prev. Vet. Med..

[204] Sojka W.J., Wray C., Shreeve J.E., Bell J.C. (1983). The incidence of salmonella infection in sheep in England and Wales, 1975 to 1981. Br. Vet. J..

[205] Methner U., Moog U. (2018). Occurrence and characterisation of Salmonella enterica subspecies diarizonae serovar 61. K: 1, 5, (7) in sheep in the federal state of Thuringia, Germany. BMC Vet. Res..

[206] Harp J.A., Myers L.L., Rich J.E., Gates N.L. (1981). Role of Salmonella arizonae and other infective agents in enteric disease of lambs. Am. J. Vet. Res..

[207] Pritchard J.A. (1990). Salmonella arizonae in sheep. Can. Vet. J. La Rev. Vet. Can..

[208] Sandberg M., Alvseike O., Skjerve E. (2002). The prevalence and dynamics of Salmonella enterica IIIb 61:k:1,5,(7) in sheep flocks in Norway. Prev. Vet. Med..

[209] McOrist S., Miller G.T. (1981). Salmonellosis in transported feral goats. Aust. Vet. J..

[210] Duffy L., Barlow R., Fegan N., Vanderlinde P. (2009). Prevalence and serotypes of Salmonella associated with goats at two Australian abattoirs. Lett. Appl. Microbiol..

[211] Al-Habsi K., Jordan D., Harb A., Laird T., Yang R., O’Dea M., Jacobson C., Miller D.W., Ryan U., Abraham S. (2018). Salmonella enterica isolates from Western Australian rangeland goats remain susceptible to critically important antimicrobials. Sci. Rep..

[212] Otesile E.B., Ahmed G., Adetosoye A.I. (1990). Experimental infection of Red Sokoto goats with Salmonella typhimurium. Rev. D’elevage De Med. Vet. Des. Pays Trop..

[213] Sharma A.K., Tripathi B.N., Verma J.C., Parihar N.S. (2001). Experimental Salmonella enterica subspecies enterica serovar Typhimurium infection in Indian goats. Clinical, serological, bacteriological and pathological studies. Small Rumin. Res..

[214] Bellinzoni R.C., Blackhall J., Terzolo H.R., Moreira A.R., Auza N., Mattion N., Micheo G.L., La Torre J.L., Scodeller E.A. (1990). Microbiology of diarrhoea in young beef and dairy calves in Argentina. Rev. Argent Microbiol..

[215] Hall G.A., Reynolds D.J., Parsons K.R., Bland A.P., Morgan J.H. (1988). Pathology of calves with diarrhoea in southern Britain. Res. Vet. Sci..

[216] Almawly J., Prattley D., French N.P., Lopez-Villalobos N., Hedgespeth B., Grinberg A. (2013). Utility of halofuginone lactate for the prevention of natural cryptosporidiosis of calves, in the presence of co-infection with rotavirus and Salmonella Typhimurium. Vet. Parasitol..

[217] Cama V.A., Mathison B.A. (2015). Infections by Intestinal Coccidia and *Giardia duodenalis*. Clin. Lab. Med..

[218] Bartelt L.A., Sartor R. (2015). Balfour Advances in understanding *Giardia*. Determinants and mechanisms of chronic sequelae. F1000prime Rep..

[219] Geurden T., Vercruysse J., Claerebout E. (2010). Is Giardia a significant pathogen in production animals?. Exp. Parasitol..

[220] O’Handley R.M., Cockwill C., McAllister T.A., Jelinski M., Morck D.W., Olson M.E. (1999). Duration of naturally acquired giardiosis and cryptosporidiosis in dairy calves and their association with diarrhea. J. Am. Vet. Med. Assoc..

[221] Aloisio F., Filippini G., Antenucci P., Lepri E., Pezzotti G., Cacciò S.M., Pozio E. (2006). Severe weight loss in lambs infected with *Giardia duodenalis* assemblage B. Vet. Parasitol..

[222] Sweeny J.P.A., Ryan U.M., Robertson I.D., Jacobson C. (2011). *Cryptosporidium* and Giardia associated with reduced lamb carcase productivity. Vet. Parasitol..

[223] Geurden T., Vanderstichel R., Pohle H., Ehsan A., von Samson-Himmelstjerna G., Morgan E.R., Camuset P., Capelli G., Vercruysse J., Claerebout E. (2012). A multicentre prevalence study in Europe on *Giardia duodenalis* in calves, with molecular identification and risk factor analysis. Vet. Parasitol..

[224] Certad G., Viscogliosi E., Chabé M., Cacciò S.M. (2017). Pathogenic Mechanisms of *Cryptosporidium* and Giardia. Trends Parasitol..

[225] Yang R., Jacobson C., Gordon C., Ryan U. (2009). Prevalence and molecular characterisation of *Cryptosporidium* and Giardia species in pre-weaned sheep in Australia. Vet. Parasitol..

[226] Ng J., Yang R., McCarthy S., Gordon C., Hijjawi N., Ryan U. (2011). Molecular characterization of *Cryptosporidium* and Giardia in pre-weaned calves in Western Australia and New South Wales. Vet. Parasitol..

[227] Dixon B., Parrington L., Cook A., Pintar K., Pollari F., Kelton D., Farber J. (2011). The potential for zoonotic transmission of *Giardia duodenalis* and *Cryptosporidium* spp. from beef and dairy cattle in Ontario, Canada. Vet. Parasitol..

[228] Coklin T., Farber J., Parrington L., Dixon B. (2007). Prevalence and molecular characterization of *Giardia duodenalis* and *Cryptosporidium* spp. in dairy cattle in Ontario, Canada. Vet. Parasitol..

[229] Santín M., Trout J.M., Fayer R. (2007). Prevalence and molecular characterization of *Cryptosporidium* and Giardia species and genotypes in sheep in Maryland. Vet. Parasitol..

[230] Lichtmannsperger K., Hinney B., Joachim A., Wittek T. (2019). Molecular characterization of Giardia intestinalis and *Cryptosporidium parvum* from calves with diarrhoea in Austria and evaluation of point-of-care tests. Comp. Immunol. Microbiol. Infect. Dis..

[231] Wade S.E., Mohammed H.O., Schaaf S.L. (2000). Prevalence of Giardia sp. Cryptosporidium parvum and Cryptosporidium andersoni (syn. C. muris) correction of Cryptosporidium parvum and Cryptosporidium muris (C. andersoni) in 109 dairy herds in five counties of southeastern New York. Vet. Parasitol..

[232] Mendonça C., Almeida A., Castro A., Lurdes Delgado M.d., Soares S., da Costa J.M.C., Canada N. (2007). Molecular characterization of *Cryptosporidium* and *Giardia* isolates from cattle from Portugal. Vet. Parasitol..

[233] Santín M., Trout J.M., Fayer R. (2009). A longitudinal study of *Giardia duodenalis* genotypes in dairy cows from birth to 2 years of age. Vet. Parasitol..

[234] Castro-Hermida J.A., Carro-Corral C., González-Warleta M., Mezo M. (2006). Prevalence and intensity of infection of *Cryptosporidium* spp. and *Giardia duodenalis* in dairy cattle in Galicia (NW Spain). J. Vet. Med. B Infect. Dis. Vet. Public Health.

[235] Castro-Hermida J.A., Almeida A., González-Warleta M., Correia da Costa J.M., Rumbo-Lorenzo C., Mezo M. (2007). Occurrence of *Cryptosporidium parvum* and *Giardia duodenalis* in healthy adult domestic ruminants. Parasitol. Res..

[236] Huetink R.E., van der Giessen J.W., Noordhuizen J.P., Ploeger H.W. (2001). Epidemiology of *Cryptosporidium* spp. and *Giardia duodenalis* on a dairy farm. Vet. Parasitol..

[237] Trout J.M., Santín M., Greiner E., Fayer R. (2005). Prevalence and genotypes of *Giardia duodenalis* in post-weaned dairy calves. Vet. Parasitol..

[238] Becher K.A., Robertson I.D., Fraser D.M., Palmer D.G., Thompson R.C.A. (2004). Molecular epidemiology of *Giardia* and *Cryptosporidium* infections in dairy calves originating from three sources in Western Australia. Vet. Parasitol..

[239] Coklin T., Farber J.M., Parrington L.J., Coklin Z., Ross W.H., Dixon B.R. (2010). Temporal changes in the prevalence and shedding patterns of *Giardia duodenalis* cysts and *Cryptosporidium* spp. oocysts in a herd of dairy calves in Ontario. Can. Vet. J. = La Rev. Vet. Can..

[240] Xiao L., Herd R.P. (1994). Infection pattern of *Cryptosporidium* and Giardia in calves. Vet. Parasitol..

[241] Geurden T., Thomas P., Casaert S., Vercruysse J., Claerebout E. (2008). Prevalence and molecular characterisation of *Cryptosporidium* and Giardia in lambs and goat kids in Belgium. Vet. Parasitol..

[242] Bednarska M., Bajer A., Siński E. (1998). Calves as a potential reservoir of *Cryptosporidium parvum* and *Giardia* sp.. Ann. Agric. Environ. Med..

[243] Björkman C., Svensson C., Christensson B., de Verdier K. (2003). *Cryptosporidium parvum* and *Giardia intestinalis* in calf diarrhoea in Sweden. Acta Vet. Scand..

[244] Ryan U.M., Bath C., Robertson I., Read C., Elliot A., McInnes L., Traub R., Besier B. (2005). Sheep may not be an important zoonotic reservoir for *Cryptosporidium* and Giardia parasites. Appl. Environ. Microbiol..

[245] Robertson L.J., Gjerde B.K., Furuseth Hansen E. (2010). The zoonotic potential of Giardia and *Cryptosporidium* in Norwegian sheep. A longitudinal investigation of 6 flocks of lambs. Vet. Parasitol..

[246] Matsuura Y., Matsubayashi M., Nukata S., Shibahara T., Ayukawa O., Kondo Y., Matsuo T., Uni S., Furuya M., Tani H. (2017). Report of fatal mixed infection with *Cryptosporidium parvum* and Giardia intestinalis in neonatal calves. Acta Parasitol..

[247] Daugschies A., Najdrowski M. (2005). Eimeriosis in cattle. Current understanding. J. Vet. Med. B Infect. Dis. Vet. Public Health.

[248] Bangoura B., Daugschies A. (2019). Coccidiosis in Cattle. Coccidiosis in Livestock, Poultry, Companion Animals and Humans.

[249] Keeton S.T.N., Navarre C.B. (2018). Coccidiosis in Large and Small Ruminants. Vet. Clin. N. Am. Food Anim. Pract..

[250] Khodakaram-Tafti A., Hashemnia M. (2017). An overview of intestinal coccidiosis in sheep and goats. Rev. Med. Vet..

[251] Bangoura B., Mundt H., Schmäschke R., Westphal B., Daugschies A. (2011). Prevalence of Eimeria bovis and Eimeria zuernii in German cattle herds and factors influencing oocyst excretion. Parasitol. Res..

[252] Enemark H.L., Dahl J., Enemark J.M.D. (2013). Eimeriosis in Danish dairy calves--correlation between species, oocyst excretion and diarrhoea. Parasitol. Res..

[253] Lee S., Kim H., Lee H., Kim J.W., Lee Y., Chae M.J., Oh S.I., Kim J.H., Rhee M.H., Kwon O.D. (2018). Eimeria species in cattle with diarrhoea in the Republic of Korea regarding age, season and nature of diarrhoea. Vet. Rec..

[254] von Samson-Himmelstjerna G., Epe C., Wirtherle N., Heyden V.v., Welz C., Radeloff I., Beening J., Carr D., Hellmann K., Schnieder T. (2006). Clinical and epidemiological characteristics of Eimeria infections in first-year grazing cattle. Vet. Parasitol..

[255] Saratsis A., Joachim A., Alexandros S., Sotiraki S. (2011). Lamb coccidiosis dynamics in different dairy production systems. Vet. Parasitol..

[256] Saratsis A., Karagiannis I., Brozos C., Kiossis E., Tzanidakis N., Joachim A., Sotiraki S. (2013). Lamb eimeriosis. Applied treatment protocols in dairy sheep production systems. Vet. Parasitol..

[257] de Souza Rodrigues F., Cezar A.S., Menezes F.R.d., Sangioni L.A., Vogel F.S.F., de Avila Botton S. (2017). Efficacy and economic analysis of two treatment regimens using toltrazuril in lambs naturally infected with Eimeria spp. on pasture. Parasitol. Res..

[258] Koudela B., Boková A. (1998). Coccidiosis in goats in the Czech Republic. Vet. Parasitol..

[259] Dai Y.B., Liu X.Y., Liu M., Tao J.P. (2006). Pathogenic effects of the coccidium Eimeria ninakohlyakimovae in goats. Vet. Res. Commun..

[260] Ruiz A., González J.F., Rodríguez E., Martín S., Hernández Y.I., Almeida R., Molina J.M. (2006). Influence of climatic and management factors on Eimeria infections in goats from semi-arid zones. J. Vet. Med. B Infect. Dis. Vet. Public Health.

[261] Koutny H., Joachim A., Tichy A., Baumgartner W. (2012). Bovine Eimeria species in Austria. Parasitol. Res..

[262] Platzer B., Prosl H., Cieslicki M., Joachim A. (2005). Epidemiology of Eimeria infections in an Austrian milking sheep flock and control with diclazuril. Vet. Parasitol..

[263] Dittmar K., Mundt H., Grzonka E., Daugschies A., Bangoura B. (2010). Ovine coccidiosis in housed lambs in Saxony-Anhalt (central Germany). Berl. Und Munch. Tierarztl. Wochenschr..

[264] Mitchell E.S.E., Smith R.P., Ellis-Iversen J. (2012). Husbandry risk factors associated with subclinical coccidiosis in young cattle. Vet. J..

[265] Seppä-Lassila L., Orro T., Lassen B., Lasonen R., Autio T., Pelkonen S., Soveri T. (2015). Intestinal pathogens, diarrhoea and acute phase proteins in naturally infected dairy calves. Comp. Immunol. Microbiol. Infect. Dis..

[266] Juszczak M., Sadowska N., Udała J. (2019). Parasites of the digestive tract of sheep and goats from organic farms in Western Pomerania, Poland. Ann. Parasitol..

[267] Lassen B., Viltrop A., Raaperi K., Järvis T. (2009). Eimeria and *Cryptosporidium* in Estonian dairy farms in regard to age, species, and diarrhoea. Vet. Parasitol..

[268] Peter G.S., Gitau G.K., Mulei C.M., Vanleeuwen J., Richards S., Wichtel J., Uehlinger F., Mainga O. (2015). Prevalence of Cryptosporidia, Eimeria, Giardia, and Strongyloides in pre-weaned calves on smallholder dairy farms in Mukurwe-ini district, Kenya. Vet. World.

[269] Cornelissen A.W.C.A., Verstegen R., van den Brand H., Perie N.M., Eysker M., Lam T.J.G.M., Pijpers A. (1995). An observational study of Eimeria species in housed cattle on Dutch dairy farms. Vet. Parasitol..

[270] Sweeny J.P.A., Robertson I.D., Ryan U.M., Jacobson C., Woodgate R.G. (2012). Impacts of naturally acquired protozoa and strongylid nematode infections on growth and faecal attributes in lambs. Vet. Parasitol..

[271] Jäger M., Gauly M., Bauer C., Failing K., Erhardt G., Zahner H. (2005). Endoparasites in calves of beef cattle herds. Management systems dependent and genetic influences. Vet. Parasitol..

[272] Burke J.M., Miller J.E., Mosjidis J.A., Terrill T.H. (2012). Use of a mixed sericea lespedeza and grass pasture system for control of gastrointestinal nematodes in lambs and kids. Vet. Parasitol..

[273] Coelho W.M.D., do Amarante A.F.T., Bresciani K.D.S. (2012). Occurrence of gastrointestinal parasites in goat kids. Rev. Bras. De Parasitol. Vet. Braz. J. Vet. Parasitol. Orgao Do Col. Bras. De Parasitol. Vet..

[274] Roeber F., Jex A.R., Campbell A.J.D., Nielsen R., Anderson G.A., Stanley K.K., Gasser R.B. (2012). Establishment of a robotic, high-throughput platform for the specific diagnosis of gastrointestinal nematode infections in sheep. Int. J. Parasitol..

[275] Holm S.A., Sörensen C.R.L., Thamsborg S.M., Enemark H.L. (2014). Gastrointestinal nematodes and anthelmintic resistance in Danish goat herds. Parasite.

[276] Avramenko R.W., Redman E.M., Lewis R., Bichuette M.A., Palmeira B.M., Yazwinski T.A., Gilleard J.S. (2017). The use of nemabiome metabarcoding to explore gastro-intestinal nematode species diversity and anthelmintic treatment effectiveness in beef calves. Int. J. Parasitol..

[277] Busato A., Lentze T., Hofer D., Burnens A., Hentrich B., Gaillard C. (1998). A case control study of potential enteric pathogens for calves raised in cow-calf herds. Zent. Veterinarmed. Reihe B J. Vet. Med. Ser. B.

[278] Gorsich E.E., Ezenwa V.O., Jolles A.E. (2014). Nematode-coccidia parasite co-infections in African buffalo. Epidemiology and associations with host condition and pregnancy. Int. J. Parasitol. Parasites Wildl..

[279] Knowles S.C.L., Fenton A., Petchey O.L., Jones T.R., Barber R., Pedersen A.B. (2013). Stability of within-host-parasite communities in a wild mammal system. Proc. Biol. Sci..

[280] Clerc M., Fenton A., Babayan S.A., Pedersen A.B. (2019). Parasitic nematodes simultaneously suppress and benefit from coccidian coinfection in their natural mouse host. Parasitology.

[281] Lass S., Hudson P.J., Thakar J., Saric J., Harvill E., Albert R., Perkins S.E. (2013). Generating super-shedders. Co-infection increases bacterial load and egg production of a gastrointestinal helminth. J. R. Soc. Interface.

[282] Clerc M., Devevey G., Fenton A., Pedersen A.B. (2018). Antibodies and coinfection drive variation in nematode burdens in wild mice. Int. J. Parasitol..

[283] Morin M., Lariviee S., Lallier R., Begin M.E., Ethier R., Roy R.S., Tremblay A. (1980). Diarrhoea of newborn calves. II. Agents responsible for the disease on Quebec dairy farms. Med. Vet. Quebec..

[284] Reynolds D.J., Morgan J.H., Chanter N., Jones P.W., Bridger J.C., Debney T.G., Bunch K.J. Microbiology of calf diarrhoea in southern Britain. Vet. Rec..

[285] Salgame P., Yap G.S., Gause W.C. (2013). Effect of helminth-induced immunity on infections with microbial pathogens. Nat. Immunol..

[286] Seabloom E.W., Borer E.T., Gross K., Kendig A.E., Lacroix C., Mitchell C.E., Mordecai E.A., Power A.G. (2015). The community ecology of pathogens. Coinfection, coexistence and community composition. Ecol. Lett..

